# Polyubiquitin and ubiquitin-like signals share common recognition sites on proteasomal subunit Rpn1

**DOI:** 10.1016/j.jbc.2021.100450

**Published:** 2021-02-20

**Authors:** Andrew J. Boughton, Daoning Zhang, Rajesh K. Singh, David Fushman

**Affiliations:** Department of Chemistry and Biochemistry, Center for Biomolecular Structure and Organization, University of Maryland, College Park, Maryland, USA

**Keywords:** rpn1, proteasome, ubiquitin, UBL domain, UBA domain, CP, core particle, CSP, chemical shift perturbation, DUB, deubiquitinase, PC, proteasome/cyclosome, polyUb, polyubiquitin, RP, regulatory particle, Ub, ubiquitin, UBA, ubiquitin-associated, UBL, ubiquitin-like, UIM, ubiquitin-interacting motif, UPS, ubiquitin–proteasome system

## Abstract

Proteasome-mediated substrate degradation is an essential process that relies on the coordinated actions of ubiquitin (Ub), shuttle proteins containing Ub-like (UBL) domains, and the proteasome. Proteinaceous substrates are tagged with polyUb and shuttle proteins, and these signals are then recognized by the proteasome, which subsequently degrades the substrate. To date, three proteasomal receptors have been identified, as well as multiple shuttle proteins and numerous types of polyUb chains that signal for degradation. While the components of this pathway are well-known, our understanding of their interplay is unclear—especially in the context of Rpn1, the largest proteasomal subunit. Here, using nuclear magnetic resonance (NMR) spectroscopy in combination with competition assays, we show that Rpn1 associates with UBL-containing proteins and polyUb chains, while exhibiting a preference for shuttle protein Rad23. Rpn1 appears to contain multiple Ub/UBL-binding sites, theoretically as many as one for each of its hallmark proteasome/cyclosome repeats. Remarkably, we also find that binding sites on Rpn1 can be shared among Ub and UBL species, while proteasomal receptors Rpn1 and Rpn10 can compete with each other for binding of shuttle protein Dsk2. Taken together, our results rule out the possibility of exclusive recognition sites on Rpn1 for individual Ub/UBL signals and further emphasize the complexity of the redundancy-laden proteasomal degradation pathway.

The ubiquitin–proteasome system (UPS) is the primary pathway for regulated protein turnover in eukaryotes (1), responsible for more than 80% of intracellular proteolysis (2). Specific polyubiquitin (polyUb) chains are conjugated to substrates through an ATP-dependent enzymatic cascade and subsequently recognized by the 26S proteasome, which then degrades the substrate. These steps are implemented in conjunction with a myriad of nuanced regulatory features (1, 3, 4), not all of which will be discussed here.

The 26S proteasome is a 2.5 MDa molecular machine that is clustered into two multisubunit complexes: the 19S regulatory particle (RP) and the 20S core particle (CP), present in a 2:1 stoichiometry. The RP is responsible for substrate recognition and translocation into the cylindrical CP, which contains six proteolytic sites. Three RP subunits function as polyUb receptors: Rpn1/PSMD2 (5, 6), Rpn10/S5a (7, 8), and Rpn13/ADRM1 (9, 10). PolyUb is first anchored to one of these receptors, after which a hexameric ring of AAA-ATPases (Rpt1-6) in the CP begins unfolding the attached substrate (11). Concurrently, proteasome-associated deubiquitinases (DUBs) dismantle the polyUb chain into Ub monomers, thereby disassociating Ub from the substrate and the RP (4, 12, 13). The unraveled substrate is then transported through a narrow opening into the hollow chamber of the CP and proteolyzed into short peptides (4). This process can be repeated with another substrate.

Intriguingly, polyUb does not always directly interact with proteasomal receptors. UPS-associated shuttle proteins, which contain ubiquitin-like (UBL) and ubiquitin-associated (UBA) domains, are also heavily involved. The UBA domain of a shuttle protein binds to polyUb (14, 15), while the UBL domain can bind to Rpn1 (5, 16, 17, 18, 19, 20), Rpn10 (21), or Rpn13 (9, 20). For reasons not yet understood, these UBL-UBA proteins interact with polyUb that is already conjugated to a substrate, subsequently “shuttle” it to the proteasome, and then associate with one of the RP receptors. In fact, these shuttle proteins may exhibit stronger affinity for the proteasome than polyUb does (5, 9, 17, 20, 21). The preeminent UBL-UBA proteins are Rad23/hHR23, Dsk2/hPLIC-1/Ubiquilin-1, and Ddi1/hDDI1—although the role of Ddi1 within the UPS is debated (17, 18). Furthermore, it has been suggested that Ubp6/hUSP14, a DUB that transiently associates with Rpn1 through its UBL domain, may also function as a polyUb receptor (13).

At ∼110 kDa, Rpn1 is the largest subunit of the proteasome. Bioinformatics analyses predict Rpn1 to contain 9–11 helix–turn–helix proteasome/cyclosome (PC) repeats, each 35–40 residues long, which form a curved toroidal structure (22, 23). These PC repeats occupy the central section of Rpn1, adjoining less characterized N- and C-terminal regions (Fig. 1). The PC region of Rpn1 reportedly interacts with various polyUb species, as well as the UBL domains of Rad23, Dsk2, Ddi1 (weakly), and Ubp6 (5, 16, 17, 19, 20, 24). Although a recognition site for proteasomal signals in the central region of Rpn1 was recently identified (5, 6, 20), those studies utilized short Rpn1 constructs. Thus, detailed information regarding the quantity and specificity of binding sites for the whole of Rpn1 is less available. Notably, Rpn10 and Rpn13 from yeast each possess only one Ub/UBL recognition motif (ubiquitin-interacting motif (UIM) (7, 25) and Pru (9) domains, respectively), while Rpn1 contains nearly a dozen PC repeats (22, 23).

**Figure 1:**
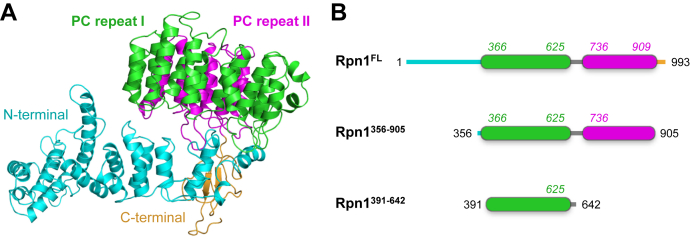
**Structure of Rpn1.***A*, structure of Rpn1 from *Saccharomyces cerevisiae* (PDB: 5A5B); the N-terminal region is cyan, the C-terminal region is *orange*, and the PC repeat regions are green and magenta. *B*, schematic of Rpn1 fragments used in this study, with the same coloring as in (*A*).

Although the UPS is the subject of intense research efforts, the apparent redundancy surrounding substrate delivery (polyUb, UBL-UBA shuttle proteins) and recognition (Rpn1, Rpn10, Rpn13) has not been clarified. While the proteasome itself is essential to cells, deletion of only one receptor or UBL-UBA shuttle protein is not lethal (5). Moreover, a significant proportion of active proteasomes do not contain Rpn10 or Rpn13, thereby demonstrating the redundant nature of UPS-mediated proteolysis (26).

Previous studies have shown that Rpn1 interacts with polyUb and UBL-UBA shuttle proteins; however, the quantity of binding sites on Rpn1—as well as their location and specificity—remains unclarified. Recent experiments demonstrated that proteasomes with concurrent mutations in all three known recognition sites (*rpn1-ARR* (5), *rpn10-uim* (8), *rpn13-pru* (5)) retain partial ability to degrade substrates (27), thereby suggesting the presence of at least one additional unidentified recognition site. Furthermore, pull-down assays have shown that Rpn1 may be able to bind the UBL domains of Rad23 and Dsk2 simultaneously (17), suggestive of multiple recognition sites on Rpn1. Here, we confirmed the physical association between Rad23, Dsk2, Ubp6, and Rpn1, both with full-length constructs and with individual domains. Intriguingly, our NMR experiments did indeed suggest the presence of more than one binding site on Rpn1. Moreover, competition studies demonstrated the multiplicity of signal recognition by Rpn1, clearly showing that Rad23, Dsk2, Ubp6, K48-linked polyUb, and K11-linked polyUb all associate with the same binding site(s) on Rpn1. These findings provide further evidence of the redundancy within the UPS and may suggest that it is actually a desirable feature.

## Results

### Rpn1 recognizes the UBL domains of Dsk2, Rad23, and Ubp6, but not Ddi1

The affinities of several UBL domains have been previously examined by SPR for full-length Rpn1 (Rpn1^FL^) and by NMR or ITC for some short Rpn1 constructs (5, 6, 17); the reported dissociation constants (K_d_) were in the low-micromolar to submicromolar range (Tables S1 and S2). These values will be referred to explicitly throughout the text. Here, we investigate two important questions in the context of signal recognition by Rpn1: (1) Does Rpn1 possess multiple UBL recognition sites, as suggested by recent findings (27)? (2) If so, is there an exclusive site for each proteasomal signal, or are the sites shared among all signals or only among some signals?

We first confirmed the interaction between Rpn1^FL^ and each of the full-length UBL-UBA proteins, as well as Ubp6. Substantial signal attenuations were observed in the NMR spectra of ^15^N-enriched Dsk2, Rad23, and Ubp6 after adding an equimolar amount of unlabeled Rpn1^FL^ (Fig. S1). Notably, the signals that disappeared predominantly corresponded to residues in the respective UBL domains, thereby supporting previous observations (5, 16, 17, 20) that Rpn1 specifically interacts with the UBL domains *in vitro*.

Attenuation of NMR signals upon binding is generally the consequence of (1) slower overall molecular tumbling due to increased size of the resulting complex; and/or (2) local fluctuations (called chemical exchange) in the electronic environment of the observed nucleus that accompany the association/dissociation events. The latter may cause severe signal broadening and disappearance when the exchange rate is on the order of the resonance frequency difference for a given nucleus in the exchanging states (28). This so-called intermediate exchange regime is usually observed for a limited subset of residues at or near the binding interface, with fluctuations typically on the submillisecond–millisecond timescale. When the chemical exchange rate is much slower than the resonance frequency difference, the slow exchange regime is observed, wherein disappearance of NMR signals corresponding to the unbound state is accompanied by the emergence of NMR signals corresponding to the bound state (29). Unlike intermediate or slow chemical exchange, a substantial increase in molecular size upon binding produces widespread signal broadening across all residues located in structured regions of the protein. The severity of this broadening depends on the size of the complex, the fraction of time each protein molecule spends in the bound state, and the fraction of protein molecules that come into contact with the ligand during the NMR measurement time (typically 100–150 ms); the former two variables determine the apparent signal linewidths. If the exchange between free and bound states happens rapidly during NMR experimentation, it is likely that most protein molecules will exhibit size-related signal broadening. However, if the dissociation rate is slow, such that the residence time in the bound state is longer than the NMR measurement time, then the fraction of protein molecules affected by this broadening will be limited by the stoichiometry of binding and the number of ligand molecules in solution. In the case when dissociation is slow and the protein concentration exceeds the concentration of available binding sites on the ligands, the excess protein molecules will not sample the bound state during the NMR measurement time, and their signals will remain visible.

It is possible that both of the aforementioned causes of signal broadening contributed to the disappearance of NMR signals in the presence of Rpn1^FL^. However, because this was a widespread effect across entire UBL domains (Figs. S2 and S3), we conclude that size-related signal broadening was the predominant factor. The molecular weight of Rpn1 complexes with the aforementioned moieties is expected to be at least ∼150 kDa for the full-length proteins and ∼120 kDa for the UBL domains, well above the typical molecular weight threshold for detectable NMR signals at the magnetic fields utilized here. Furthermore, the reported dissociation rates of Rpn1^FL^ with each of Dsk2, Rad23, and Ubp6 are so slow that the residence time in the complex (6–15 s) greatly exceeds the NMR measurement time (17) (Table S2), thus ruling out intermediate exchange as the cause of signal disappearance.

**Figure 2:**
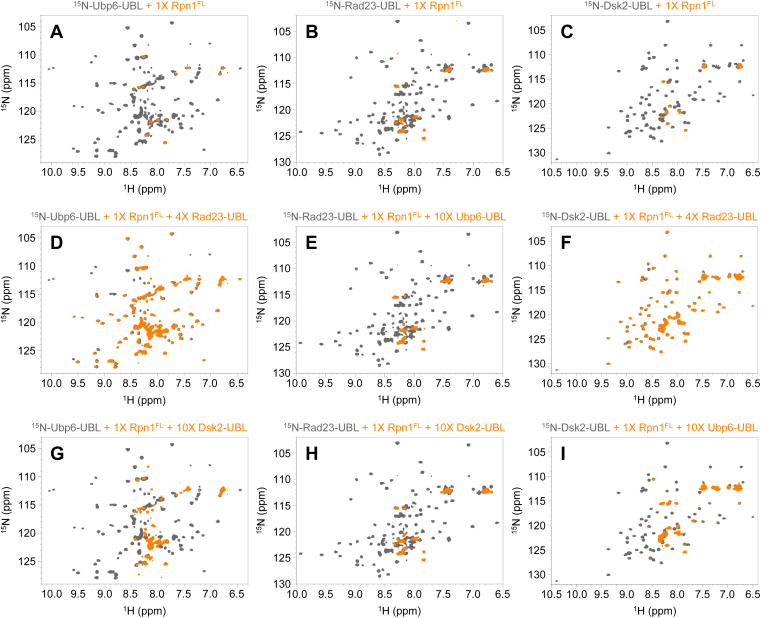
**Rad23 outcompetes Dsk2 and Ubp6 for binding of Rpn1.** Overlaid ^1^H-^15^N NMR spectra of: (*A*) ^15^N-Ubp6-UBL (*gray*), ^15^N-Ubp6-UBL plus 1× Rpn1^FL^ (*orange*); (*B*) ^15^N-Rad23-UBL (*gray*), ^15^N-Rad23-UBL plus 1× Rpn1^FL^ (*orange*); (*C*) ^15^N-Dsk2-UBL (*gray*), ^15^N-Dsk2-UBL plus 1× Rpn1^FL^ (*orange*); (*D*) ^15^N-Ubp6-UBL (*gray*), ^15^N-Ubp6-UBL plus 1× Rpn1^FL^ and 4× Rad23-UBL (*orange*); (*E*) ^15^N-Rad23-UBL (*gray*), ^15^N-Rad23-UBL plus 1× Rpn1^FL^ and 10× Ubp6-UBL (*orange*); (*F*) ^15^N-Dsk2-UBL (*gray*), ^15^N-Dsk2-UBL plus 1× Rpn1^FL^ and 4× Rad23-UBL (*orange*); (*G*) ^15^N-Ubp6-UBL (*gray*), ^15^N-Ubp6-UBL plus 1× Rpn1^FL^ and 10× Dsk2-UBL (*orange*); (*H*) ^15^N-Rad23-UBL (*gray*), ^15^N-Rad23-UBL plus 1× Rpn1^FL^ and 10× Dsk2-UBL (*orange*); (*I*) ^15^N-Dsk2-UBL (*gray*), ^15^N-Dsk2-UBL plus 1× Rpn1^FL^ and 10× Ubp6-UBL (*orange*). In (*A–I*), the concentration of the ^15^N-enriched protein was 30 μM, such that a 1× molar equivalency corresponded to 30 μM, a 4× molar equivalency corresponded to 120 μM, and a 10× molar equivalency corresponded to 300 μM.

Because we observed that Rpn1 primarily recognizes the UBL domains of Dsk2, Rad23, and Ubp6 but not the UBA domains of the former two shuttle proteins (Fig. S1), the binding experiments were repeated with only the isolated UBL domain of each protein. Substantial signal attenuations were seen in the NMR spectra of ^15^N-Dsk2-UBL, ^15^N-Rad23-UBL, and ^15^N-Ubp6-UBL in the presence of an equimolar amount of Rpn1^FL^ (Fig. 2, *A–C*). The few signals that remained visible after adding Rpn1^FL^ belonged to flexible regions and termini (Figs. S2 and S3); this behavior is consistent with size-related disappearance of NMR signals corresponding to the UBL domains of full-length Dsk2, Rad23, and Ubp6 upon addition of Rpn1^FL^ as a result of slow overall molecular tumbling of the complex. Thus, our further experiments utilize only UBLs, as they provide essentially identical results to the full-length UBL-containing proteins along with less crowded NMR spectra (compare Figs. S1 and S2).

By contrast, only negligible signal attenuations were present in the NMR spectrum of full-length ^15^N-Ddi1 after adding excess Rpn1^FL^ (Fig. S4*A*), suggesting that Rpn1 does not interact with Ddi1. Likewise, signals from ^15^N-Ddi1-UBL did not exhibit a noticeable intensity decrease upon addition of excess Rpn1^FL^ (Fig. S4*B*). We also examined the possible association of Ddi1-UBL with two shorter Rpn1 constructs (Rpn1^356-905^ and Rpn1^391-642^, to be discussed later; see Fig. 1*B*), neither of which caused notable perturbations in the signals of ^15^N-Ddi1-UBL (Fig. S4, *C* and *D*). Contrary to previous results (19), we concluded that isolated Ddi1 does not exhibit any relevant interactions with Rpn1 *in vitro*. This is not unprecedented, as the UBL domain of Ddi1 displays atypical properties for a ubiquitin-like moiety (18).

### Rpn1 likely contains multiple recognition sites that are shared among UBL domains

Since we verified that Rpn1 binds to the UBL domains of Dsk2, Rad23, and Ubp6, we next sought to characterize the multiplicity of recognition sites on Rpn1. In our aforementioned NMR experiments with ^15^N-UBL domains and Rpn1^FL^, we observed that most UBL signals disappeared by a ^15^N-UBL:Rpn1^FL^ molar ratio of 1:0.3 (Fig. S2). Given the widespread disappearance of NMR signals (Fig. S3), this effect is not indicative of intermediate exchange on the chemical shift timescale, which usually affects a small number of residues. Substoichiometric amounts of a high-molecular-weight binding partner may cause global NMR signal broadening if the exchange between free and bound states for the observed protein occurs rapidly during the NMR measurement time (30). However, based on the aforementioned slow dissociation rates for these interactions (17) (Table S2), we speculated that a more likely explanation for this observation is that Rpn1^FL^ contains multiple UBL-binding sites. A single UBL-binding site on Rpn1 (*i.e.*, 1:1 binding stoichiometry) cannot explain the absence of NMR signals that should be observed from the excess ^15^N-UBLs in the slow exchange regime.

We tested this hypothesis by recording a ^1^H-^15^N HSQC spectrum of a sample containing an equimolar mixture of ^15^N-Rad23-UBL, ^15^N-Dsk2-UBL, and ^15^N-Ubp6-UBL (Fig. S5*A*). We added an equimolar amount of Rpn1^FL^ to this sample and observed widespread signal attenuation (Fig. S5*B*), even though three UBLs were present per Rpn1 moiety, consistent with the presence of multiple binding sites on Rpn1. After addition of each ^15^N-UBL up to a threefold molar excess of Rpn1^FL^ (3X ^15^N-Rad23-UBL, 3X ^15^N-Dsk2-UBL, and 3X ^15^N-Ubp6-UBL; resulting in a ninefold effective ^15^N-UBLs molar excess), many signals became visible again (Fig. S5*C*). We compared this final spectrum with that of each ^15^N-UBL alone and noticed that the majority of reappearing signals corresponded to ^15^N-Ubp6-UBL (Fig. S5*D*). This observation suggests two possibilities: (1) there is only one binding site for Ubp6-UBL on Rpn1, which is already saturated upon threefold addition of ^15^N-Ubp6-UBL, leading to excess ^15^N-Ubp6-UBL in solution and the reappearance of its corresponding NMR signals; and/or (2) the Ubp6-UBL binding site(s) on Rpn1 is shared among all UBLs, so addition of the other UBLs results in the displacement of ^15^N-Ubp6-UBL, hence the return of its corresponding NMR signals.

To differentiate between these two possibilities, we performed a series of competition experiments. As stated earlier, the majority of NMR signals for ^15^N-Ubp6-UBL attenuated upon addition of an equimolar amount of Rpn1^FL^ (Fig. 2*A*). However, adding excess Rad23-UBL to this sample resulted in the reemergence of ^15^N-Ubp6-UBL signals (Fig. 2*D*), indicating that Rad23-UBL can displace Ubp6-UBL from the binding site(s) on Rpn1. We also performed the reverse experiment: excess Ubp6-UBL was added to a sample containing an equimolar mixture of ^15^N-Rad23-UBL and Rpn1^FL^, yet the signals from ^15^N-Rad23-UBL did not reappear, even at a tenfold excess of Ubp6-UBL (compare Fig. 2, *B* and *E*). Thus, Ubp6-UBL cannot displace Rad23-UBL from Rpn1. These data suggest that Rad23-UBL and Ubp6-UBL bind to the same site(s) on Rpn1, which contradicts the previous report of a binding site on Rpn1 that is exclusive to Ubp6 and unable to recognize Rad23 (5).

We repeated this competition experiment with the other combinations of UBLs. Adding excess Rad23-UBL to an equimolar mixture of ^15^N-Dsk2-UBL and Rpn1^FL^ rescued the original ^15^N-Dsk2-UBL signals (compare Fig. 2, *C* and *F*). However, adding excess Dsk2-UBL to an equimolar mixture of ^15^N-Rad23-UBL and Rpn1^FL^ did not bring back any ^15^N-Rad23-UBL signals (compare Fig. 2, *B* and *H*). This is an indication that Rad23-UBL outcompetes Dsk2-UBL for binding to Rpn1, thereby suggesting that Rad23-UBL and Dsk2-UBL also bind to the same site(s) on Rpn1.

Finally, we added excess Dsk2-UBL to an equimolar mixture of ^15^N-Ubp6-UBL and Rpn1^FL^ (compare Fig. 2, *A* and *G*), as well as excess Ubp6-UBL to an equimolar mixture of ^15^N-Dsk2-UBL and Rpn1^FL^ (compare Fig. 2, *C* and *I*). In both cases, signals from the original ^15^N-UBL species returned, to some extent. This suggests that Dsk2-UBL and Ubp6-UBL also share binding sites on Rpn1. From these data, we concluded that Rpn1^FL^ likely contains multiple UBL-binding sites, which are promiscuous enough to be shared among Rad23-UBL, Dsk2-UBL, and Ubp6-UBL, thus challenging the possibility that Rpn1 contains a distinct recognition site for each signal.

### Rpn10 competes with Rpn1 for binding to Dsk2, but not Rad23

**Figure 3:**
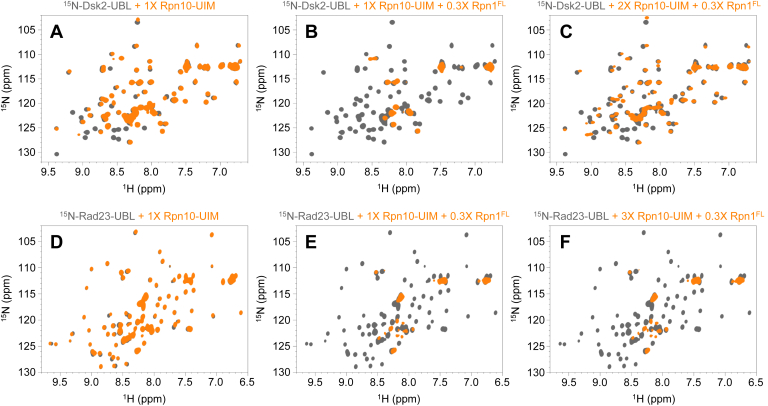
**Rpn10 competes with Rpn1 for binding to Dsk2, but not to Rad23.** Overlaid ^1^H-^15^N NMR spectra of: (*A*) ^15^N-Dsk2-UBL (*gray*), ^15^N-Dsk2-UBL plus 1× Rpn10-UIM (*orange*); (*B*) ^15^N-Dsk2-UBL (*gray*), ^15^N-Dsk2-UBL plus 1× Rpn10-UIM and 0.3× Rpn1^FL^ (*orange*); (*C*) ^15^N-Dsk2-UBL (*gray*), ^15^N-Dsk2-UBL plus 2× Rpn10-UIM and 0.3× Rpn1^FL^ (*orange*); (*D*) ^15^N-Rad23-UBL (*gray*), ^15^N-Rad23-UBL plus 1× Rpn10-UIM (*orange*); (*E*) ^15^N-Rad23-UBL (*gray*), ^15^N-Rad23-UBL plus 1× Rpn10-UIM and 0.3× Rpn1^FL^ (*orange*); (*F*) ^15^N-Rad23-UBL (*gray*), ^15^N-Rad23-UBL plus 3× Rpn10-UIM and 0.3× Rpn1^FL^ (*orange*). In (*A–F*), the concentration of the ^15^N-enriched protein was 150 μM, such that a 0.3× molar equivalency corresponded to 48 μM, a 1× molar equivalency corresponded to 150 μM, a 2× molar equivalency corresponded to 300 μM, and a 3× molar equivalency corresponded to 450 μM. UIM, ubiquitin-interacting motif.

Given the apparent competition among the proteasomal signals for binding to Rpn1, we wanted to examine if there is any competition between Rpn1 and Rpn10, one of the other proteasomal receptors. Rpn10 exhibits strong affinity for Dsk2-UBL through its UIM domain, while it binds Rad23-UBL with comparatively weak affinity and does not interact with Ubp6-UBL at all (21). Thus, ^15^N-Dsk2-UBL NMR signals shifted significantly upon equimolar addition of Rpn10-UIM (Fig. 3*A*). Adding Rpn1^FL^ to that same sample induced widespread signal attenuations (Fig. 3*B*), as a result of some ^15^N-Dsk2-UBL moieties binding to Rpn1 instead of Rpn10. However, after a further addition of Rpn10-UIM, most signals from ^15^N-Dsk2-UBL reappeared at their Rpn10-UIM-bound positions (Fig. 3*C*).

Conversely, the same experiment performed with ^15^N-Rad23-UBL instead of ^15^N-Dsk2-UBL resulted in only minimal changes once equimolar Rpn10-UIM was added (Fig. 3*D*). Subsequent addition of Rpn1^FL^ to this sample resulted in significant ^15^N-Rad23-UBL signal disappearance (Fig. 3*E*), as expected. Strikingly, even adding Rpn10-UIM to a concentration tenfold greater than that of Rpn1^FL^ did not rescue any ^15^N-Rad23-UBL signals (Fig. 3*F*). These observations indicate that Rpn10 can compete with Rpn1 for binding to Dsk2-UBL but not for binding to Rad23-UBL, at least under the conditions tested here. The notion of a preferential recognition hierarchy among UBL domains and proteasomal receptors has been suggested previously (17).

### Rpn1 PC repeat region also displays binding site multiplicity for UBL domains

To further characterize the promiscuous nature of UBL-binding sites on Rpn1, we utilized an Rpn1 construct containing only residues 356–905 (Rpn1^356-905^; see Fig. 1). This fragment was designed to cover the entire PC repeat region but is ∼50 kDa smaller than Rpn1^FL^; we hoped this size decrease would enable us to observe signal shifts upon binding, as opposed to size-related signal attenuations. It was previously shown that the first ∼200 residues of Rpn1 are not involved in signal recognition (17). Moreover, this construct exhibited improved expression and stability compared with Rpn1^FL^, which allowed additional concentration points to be sampled during competition experiments.

However, equimolar addition of Rpn1^356-905^ to ^15^N-Dsk2-UBL still triggered widespread signal attenuations (Fig. S6*A*). As with Rpn1^FL^, adding Rad23-UBL to this sample resulted in the reappearance of ^15^N-Dsk2-UBL signals (Fig. S6, *B–C*); this was apparent even at a Rad23-UBL:^15^N-Dsk2-UBL molar ratio of one (Fig. S6*B*). Conversely, a fourfold excess of Dsk2-UBL was unable to outcompete ^15^N-Rad23-UBL for binding to Rpn1^356-905^ (Fig. S6, *D–F*).

Similarly, addition of Rad23-UBL to an equimolar mixture of ^15^N-Ubp6-UBL and Rpn1^356-905^ caused a substantial return in signal intensity by a Rad23-UBL:^15^N-Ubp6-UBL molar ratio of 1, and a complete return by a ratio of 4 (Fig. S7, *A–C*). However, excess Ubp6-UBL could not outcompete ^15^N-Rad23-UBL for binding to Rpn1^356-905^ (Fig. S7, *D–F*). To ensure that this effect was not a by-product of utilizing only UBL domains instead of full-length proteins, we also added excess full-length Ubp6 to an equimolar mixture of ^15^N-Rad23-UBL with Rpn1^356-905^ and observed the same result as for Ubp6-UBL: namely, ^15^N-Rad23-UBL was not displaced (Fig. S8, *A–B*). This control experiment was important for Ubp6, as the UBP domain of Ubp6 also interacts with Rpn1 (17).

Finally, addition of Dsk2-UBL to an equimolar mixture of ^15^N-Ubp6-UBL and Rpn1^356-905^ resulted in slight signal reappearance at a Dsk2-UBL:^15^N-Ubp6-UBL molar ratio of 1 and a substantial intensity increase at a molar ratio of 4 (Fig. S9, *A–C*). A similar trend was seen when adding Ubp6-UBL to an equimolar mixture of ^15^N-Dsk2-UBL and Rpn1^356-905^ (Fig. S9, *D–F*).

Overall, these data corroborate our observations with Rpn1^FL^, wherein Rpn1 appears to contain multiple recognition sites that are shared among the UBL domains. The similar binding properties among Rpn1^FL^ and Rpn1^356-905^ were not surprising, as Rpn1^356-905^ encompasses the entire PC repeat region. However, this Rpn1 construct was still too large to allow us to monitor signal movement by NMR.

### Relaxation rates and chemical shift perturbations demonstrate competition for shared binding sites

We next sought out an even smaller Rpn1 fragment to observe residue-specific changes upon binding. This construct was comprised of residues 391–642 (Rpn1^391-642^; see Fig. 1), which is a region that contains roughly half of the PC repeats, binds UBLs strongly (6), and includes the reported Ubp6-exclusive binding site (5).

**Figure 4:**
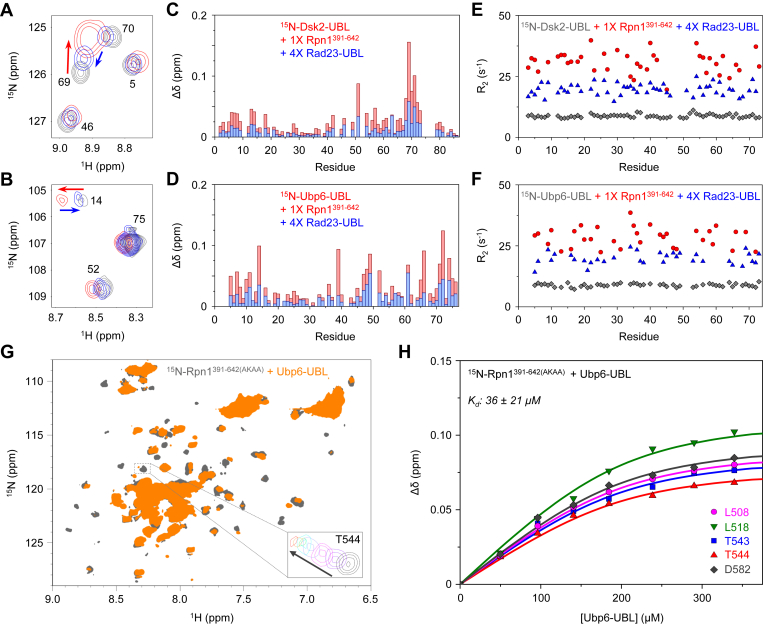
**Rpn1**^**391-642**^**also exhibits recognition site multiplicity for UBL domains.***A*, overlaid ^1^H-^15^N NMR spectra of ^15^N-Dsk2-UBL by itself (*gray*), ^15^N-Dsk2-UBL plus 1× Rpn1^391-642^ (*red*), ^15^N-Dsk2-UBL plus 1× Rpn1^391-642^ and 4× Rad23-UBL (*blue*). *B*, overlaid ^1^H-^15^N NMR spectra of ^15^N-Ubp6-UBL by itself (*gray*), ^15^N-Ubp6-UBL plus 1× Rpn1^391-642^ (*red*), ^15^N-Ubp6-UBL plus 1× Rpn1^391-642^ and 4× Rad23-UBL (*blue*). *C*, Residue-specific CSPs (Δδ, compared to ^15^N-Dsk2-UBL by itself) for ^15^N-Dsk2-UBL plus 1× Rpn1^391-642^ (*red bars*), ^15^N-Dsk2-UBL plus 1× Rpn1^391-642^ and 4× Rad23-UBL (*blue bars*). *D*, Residue-specific CSPs (Δδ, compared to ^15^N-Ubp6-UBL by itself) for ^15^N-Ubp6-UBL plus 1× Rpn1^391-642^ (*red bars*), ^15^N-Ubp6-UBL plus 1× Rpn1^391-642^ and 4× Rad23-UBL (*blue bars*). *E*, ^15^N R_2_ rates for ^15^N-Dsk2-UBL by itself (*gray*), ^15^N-Dsk2-UBL plus 1× Rpn1^391-642^ (*red*), ^15^N-Dsk2-UBL plus 1× Rpn1^391-642^ and 4× Rad23-UBL (*blue*). *F*, ^15^N R_2_ rates for ^15^N-Ubp6-UBL by itself (*gray*), ^15^N-Ubp6-UBL plus 1× Rpn1^391-642^ (*red*), ^15^N-Ubp6-UBL plus 1× Rpn1^391-642^ and 4× Rad23-UBL (*blue*). In (*A–F*), the concentration of the ^15^N-enriched protein was 150 μM, such that a 1× molar equivalency corresponded to 150 μM and a 4× molar equivalency corresponded to 600 μM. *G*, Overlaid ^1^H-^15^N NMR spectra of ^15^N-Rpn1^391-642(AKAA)^ (*gray*) and ^15^N-Rpn1^391-642(AKAA)^ plus equimolar Ubp6-UBL (*orange*); as a representative example, the signal of T544 after each addition of Ubp6-UBL is shown in the inset. *H*, NMR titration CSPs (Δδ) for select residues in ^15^N-Rpn1^391-642(AKAA)^ as a function of Ubp6-UBL concentration. The curves are a result of fitting the experimental data to a single-site binding model.

Excitingly, addition of unlabeled Rpn1^391-642^ to ^15^N-Dsk2-UBL or ^15^N-Ubp6-UBL produced significant and gradual signal shifts with only minor signal attenuations (Fig. 4, *A* and *B*), an indication that such interactions exhibit fast exchange kinetics on the NMR chemical shift timescale. Therefore, we were able to track signal movement throughout the NMR experiments, which allowed us to utilize chemical shift perturbations (CSPs) to identify residues in the UBL domains affected by binding and to quantify binding-induced changes in transverse ^15^N spin-relaxation rates (R_2_) on a per-residue basis.

Amide signals of ^15^N-Dsk2-UBL exhibited substantial CSPs after an equimolar amount of Rpn1^391-642^ was added (Fig. 4*C*, *red*), while the average ^15^N R_2_ increased from 8.8 ± 0.7 s^-1^ to 30.9 ± 4.3 s^-1^ (Fig. 4*E*), indicative of a large increase in the overall size as a result of complex formation upon binding. Note that the observed increase in R_2_, reflecting slower overall tumbling, is generally consistent with the 3.5-fold larger molecular weight of the complex compared with the isolated UBL. After addition of excess Rad23-UBL, the CSPs decreased as ^15^N-Dsk2-UBL signals returned close to their unbound location (Fig. 4*C*, *blue*), while the average R_2_ dropped to 19.7 ± 2.3 s^-1^ (Fig. 4*E*), demonstrating displacement of ^15^N-Dsk2-UBL by Rad23-UBL.

A similar trend was observed for ^15^N-Ubp6-UBL: signals shifted significantly in the presence of equimolar Rpn1^391-642^, but returned back toward their unbound positions upon addition of excess Rad23-UBL (Fig. 4*D*). Moreover, the average ^15^N R_2_ for ^15^N-Ubp6-UBL rose from 8.9 ± 0.7 s^-1^ to 28.9 ± 4.4 s^-1^ after Rpn1^391-642^ was added, but decreased to 19.8 ± 2.4 s^-1^ in the presence of excess Rad23-UBL (Fig. 4*F*). The striking similarity in signal behavior and R_2_ values between both sets of experiments suggests that Dsk2-UBL and Ubp6-UBL exhibit analogous binding modes for Rpn1^391-642^. Notably, all three UBLs retain the ability to share the same binding site(s), even on this truncated Rpn1 construct.

This observation for Rpn1^391-642^ was surprising—especially in the context of Ubp6—since it was previously reported that introducing L430A, D431K, Q434A, and Q435A (“AKAA”) mutations in Rpn1 resulted in loss of binding to Ubp6 but not Rad23 (5), thereby suggesting the presence of a Ubp6-exclusive binding site in this region. To investigate this discrepancy further, we replicated these AKAA mutations in our Rpn1 construct. However, in our hands, addition of unlabeled Ubp6-UBL to ^15^N-Rpn1^391-642(AKAA)^ still produced significant signal perturbations (Fig. 4*G*), indicative of binding. The K_d_ for this interaction was quantified as 36 ± 21 μM (Fig. 4*H*), agreeing well with the published value of 40 ± 31 μM (6) for Rpn1^391-642^. To independently verify the authenticity of these results, we performed our own control titration experiment with ^15^N-Rpn1^391-642^ and Ubp6-UBL. In this case, the NMR spectra were remarkably similar to those of ^15^N-Rpn1^391-642(AKAA)^ with Ubp6-UBL (Fig. S10*A*), while the calculated K_d_ of 43 ± 22 μM (Fig. S10*B*) was comparable with the aforementioned values. Furthermore, the perturbed residues in ^15^N-Rpn1^391-642(AKAA)^ corresponded to the same region that reportedly interacts with Rad23-UBL (5).

Notably, addition of equimolar Rpn1^391-642^ to ^15^N-Rad23-UBL still resulted in widespread signal attenuations—with no evidence of residue-specific line broadening at low Rpn1^391-642^ concentrations (Fig. S3*C*)—even though the total expected complex size was only ∼36 kDa, within the acceptable range for NMR (Fig. S8, *C–F*). It is possible that binding of Rad23-UBL to Rpn1 induces oligomerization, thereby causing the resulting complex to be adversely large. Rpn1 is prone to aggregation (5, 6), likely as a consequence of its high helical content.

As a control, we added excess Ubp6-UBL to an equimolar mixture of ^15^N-Rad23-UBL and Rpn1^391-642^; as with the other Rpn1 fragments, signals from ^15^N-Rad23-UBL did not return (Fig. S8, *C* and *D*). Adding excess full-length Ubp6 instead of Ubp6-UBL produced the same result (Fig. S8, *E–F*). These observations suggested that Rpn1^391-642^ exhibits similar binding preferences to those of the longer Rpn1 constructs. Therefore, we did not pursue experiments with ^15^N-Rad23-UBL and Rpn1^391-642^ any further, as they only allowed us to track signal intensity—not signal movement—which we have already characterized twice. Taken together, these data indicate that Rad23-UBL, Dsk2-UBL, and Ubp6-UBL promiscuously bind to the same site(s) on Rpn1^391-642^, with no evidence of a binding site exclusive to only one UBL.

### Polyubiquitin also interacts with UBL-binding sites on Rpn1

Historically, polyUb chains linked through K48 have been considered the canonical signal for proteasomal degradation. However, truncated Rpn1 appears to preferentially recognize the UBL domains of shuttle proteins (especially Rad23) over K48-linked polyUb (5, 6). To further characterize this ostensibly redundant relationship, we performed competition experiments with K48-linked Ub_2_.

**Figure 5:**
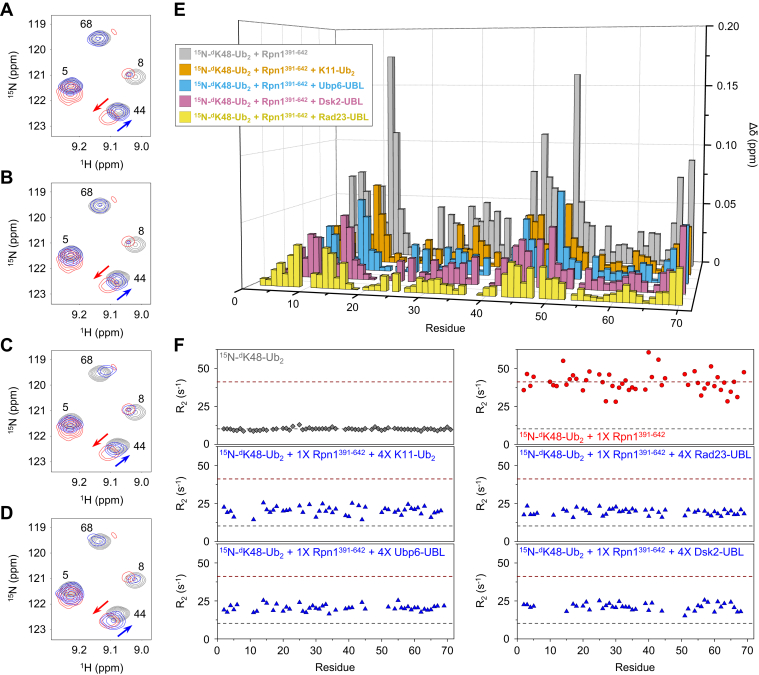
**Rad23, Dsk2, Ubp6, and K11-linked Ub**_**2**_**outcompete K48-linked Ub**_**2**_**for binding of Rpn1.***A–D*, overlaid ^1^H-^15^N NMR spectra of ^15^N-^d^K48-Ub_2_ by itself (*gray*), ^15^N-^d^K48-Ub_2_ plus 1× Rpn1^391-642^ (*red*), ^15^N-^d^K48-Ub_2_ plus 1× Rpn1^391-642^ and each of the following competitors (*blue*): (*A*) 4× Rad23-UBL; (*B*) 4× Dsk2-UBL; (*C*) 4× Ubp6-UBL; (*D*) 4× K11-Ub_2_. *E*, Residue-specific CSPs (Δδ, compared with ^15^N-^d^K48-Ub_2_ by itself) for ^15^N-^d^K48-Ub_2_ plus 1× Rpn1^391-642^ (*gray bars*), ^15^N-^d^K48-Ub_2_ plus 1× Rpn1^391-642^ and each of the following competitors: 4× Rad23-UBL (*yellow bars*); 4× Dsk2-UBL (*pink bars*); 4× Ubp6-UBL (*blue bars*); 4× K11-Ub_2_ (*orange bars*). *F*, ^15^N R_2_ rates, clockwise from top left, for ^15^N-^d^K48-Ub_2_ by itself (*gray*), ^15^N-^d^K48-Ub_2_ plus 1× Rpn1^391-642^ (*red*), ^15^N-^d^K48-Ub_2_ plus 1× Rpn1^391-642^ and each of the following competitors (*blue*): 4× Rad23-UBL; 4× Dsk2-UBL; 4× Ubp6-UBL; 4× K11-Ub_2_. *Dashed lines*, which serve as a visual aid, indicate average ^15^N R_2_ rates for ^15^N-^d^K48-Ub_2_ by itself (*gray*) and ^15^N-^d^K48-Ub_2_ plus 1× Rpn1^391-642^ (*red*). In (*A–F*), the concentration of ^15^N-^d^K48-Ub_2_ was 150 μM, such that a 1× molar equivalency corresponded to 150 μM and a 4× molar equivalency corresponded to 600 μM.

Amide signals from K48-linked Ub_2_ with the distal (lysine-accepting) Ub ^15^N-enriched (^15^N-^d^K48-Ub_2_) shifted significantly after equimolar addition of Rpn1^391-642^ (Fig. 5, *A–E*), with a concomitant increase in the average ^15^N R_2_ value from 10.1 ± 0.8 s^-1^ for ^15^N-^d^K48-Ub_2_ alone to 40.9 ± 6.7 s^-1^ in the presence of Rpn1^391-642^ (Fig. 5*F*). Note that these R_2_ values are larger than those exhibited by ^15^N-Dsk2-UBL and ^15^N-Ubp6-UBL due to the bigger size of ^15^N-^d^K48-Ub_2_. Next, we separately added a fourfold molar excess of each UBL to equimolar mixtures of ^15^N-^d^K48-Ub_2_ and Rpn1^391-642^. In all three cases, CSPs decreased significantly as ^15^N-^d^K48-Ub_2_ signals returned close to their original location (Fig. 5*E*), while average R_2_ values declined to 19.4 ± 1.9 s^-1^, 21.1 ± 2.4 s^-1^, and 20.4 ± 2.1 s^-1^ after addition of excess Rad23-UBL, Dsk2-UBL, and Ubp6-UBL, respectively (Fig. 5*F*). Remarkably, these data indicate that Rpn1 does not only promiscuously bind UBLs—in fact, the same binding sites on Rpn1 are shared among UBLs and K48-linked polyUb.

After the recent finding that Rpn1^391-642^ prefers K11-linked Ub_2_ over K48-linked Ub_2_, with respective K_d_ values of ∼28 μM and ∼123 μM (24), we performed an additional competition experiment. Adding a fourfold molar excess of K11-linked Ub_2_ to an equimolar mixture of ^15^N-^d^K48-Ub_2_ and Rpn1^391-642^ resulted in diminished CSPs and a reduced average ^15^N R_2_ of 20.4 ± 2.8 s^-1^ (Fig. 5, *E* and *F*), similar to the effect seen after adding UBLs. While it is not surprising—given the aforementioned affinities (24)—that K11-linked Ub_2_ outcompetes K48-linked Ub_2_ for binding of Rpn1^391-642^, this result also demonstrates that K11-linked Ub_2_ and K48-linked Ub_2_ bind to the same site(s) on Rpn1^391-642^. In summary, the data presented here suggest that Rad23, Dsk2, Ubp6, K48-linked polyUb, and K11-linked polyUb all share common recognition sites on Rpn1, with no evidence of an exclusive site for each signal.

## Discussion

Although numerous studies have examined the apparently redundant relationship between Rpn1, polyUb, UBL-UBA shuttle proteins, and other UBL-containing proteins, the quantity and specificity of binding sites on full-length Rpn1 remain poorly characterized. Here, we showed that Rpn1 appears to contain multiple binding sites, which preferentially associate with the UBL domain of Rad23 over other proteasomal signals. Although Rpn1 is expected to also recognize Ddi1 based on yeast two-hybrid studies (19), we did not detect binding by NMR, even though all of our Rpn1 constructs contained the putative Ddi1-binding site. This agrees with pull-down and SPR experiments that likewise found little evidence of an association between Rpn1 and Ddi1 (17). Additionally, our competition studies demonstrated that Rad23, Dsk2, Ubp6, K48-linked polyUb, and K11-linked polyUb—the primary targets of proteasomal receptors—all share the same recognition site(s) on Rpn1.

Our initial experiments with Rpn1^FL^ indicated the presence of multiple UBL-binding sites. This was demonstrated by the widespread disappearance of ^15^N-UBL signals after adding Rpn1^FL^ at an ^15^N-UBL:Rpn1^FL^ molar ratio of 1:0.3. Moreover, NMR signals from an equimolar mixture of the three ^15^N-UBLs and Rpn1^FL^ did not reappear until a threefold excess of each of the ^15^N-UBLs was reached; the effective ^15^N-UBLs:Rpn1^FL^ molar ratio at this point was 9:1, suggesting that Rpn1 may possess multiple UBL-binding sites. This result is physically cogent, as Rpn1 contains at least nine PC repeats, each of which may potentially be able to function as a recognition site.

Competition studies showed that neither Dsk2-UBL nor Ubp6-UBL was able to displace Rad23-UBL from Rpn1^FL^—even at a tenfold greater molar ratio—while Rad23-UBL could efficiently outcompete Dsk2-UBL and Ubp6-UBL; this observation was also noted with Rpn1^356-905^ (which contains the entire PC repeat region). Meanwhile, Dsk2-UBL and Ubp6-UBL were able to partially outcompete each other for binding to Rpn1^FL^ and Rpn1^356-905^; their similar capabilities for displacing the other UBL suggest that they have comparable binding modes for Rpn1. This premise was corroborated by the resemblance in signal behavior and R_2_ rates for each UBL in the presence of equimolar Rpn1^391-642^ and with an excess of the other UBL. We speculate that Rad23-UBL can displace Dsk2-UBL and Ubp6-UBL—but not vice versa—from Rpn1 due to a stronger binding affinity with Rpn1; this suggestion agrees with K_d_ values for Rpn1^391-642^ (Table S1), but is somewhat inconsistent with K_d_ values for Rpn1^FL^ (Table S2). Even though Rad23 appears to be the preferred recognition signal for Rpn1, our data show that Rad23 associates with the binding site(s) for Dsk2 and Ubp6, yet the possibility of a site exclusive to Rad23—which Dsk2 and Ubp6 cannot interact with—has not been ruled out.

Likewise, competition experiments with Rpn1^391-642^ (which contains half of the PC repeat region) clearly showed that all three UBLs can share binding sites, although the results were not as striking as for the larger Rpn1 constructs. For example, equimolar addition of Rpn1^356-905^ to ^15^N-Dsk2-UBL or ^15^N-Ubp6-UBL caused widespread signal attenuations, yet a subsequent equimolar addition of Rad23-UBL substantially rescued those signals, which returned to essentially original intensity levels at a fourfold excess of Rad23-UBL. However, the same experiment with Rpn1^391-642^ instead of Rpn1^356-905^ exhibited a ∼30% decrease in CSP and R_2_ values at a Rad23-UBL:^15^N-UBL molar ratio of 1 and only a ∼70% decrease at a ratio of 4 (Fig. 4, *C–F*). Thus, it appears that signals did not completely come back to their starting position or baseline intensity in the case of Rpn1^391-642^, perhaps indicating that this shorter Rpn1 fragment contains a binding site biased—but not exclusive—to Dsk2-UBL and/or Ubp6-UBL. The fact that these R_2_ values did not fully return to those of the free UBLs reflects the incomplete displacement of Dsk2-UBL and Ubp6-UBL by Rad23-UBL, which is also evident from the residual CSPs; we speculate that Dsk2-UBL and Ubp6-UBL are still able to spend a fraction of time in complex with Rpn1^391-642^, thereby resulting in higher R_2_ values than for the unbound state. Note that unlike binding of the full-length UBL-containing proteins or of their respective UBL domains to Rpn1^FL^, binding of Dsk2-UBL and Ubp6-UBL to Rpn1^391-642^ is in the fast exchange regime on the NMR timescale—comparing these observations made under different chemical exchange regimes is not straightforward. It is worth repeating that this region of Rpn1 purportedly contains the Ubp6-exclusive recognition site (5); nonetheless, we observed no effect of the AKAA mutations in Rpn1^391-642^, which were intended to specifically disrupt binding of Ubp6-UBL.

We have previously shown that Rpn1^391-642^ exhibits roughly fourfold stronger affinity for K11-linked Ub_2_ than for K48-linked Ub_2_ (24). Since polyUb linked through K48 is considered the quintessential signal for proteasomal degradation, we wanted to investigate this relationship in greater detail. Remarkably, K48-linked Ub_2_ was consistently outcompeted for binding of Rpn1^391-642^, thereby suggesting that recognition sites on Rpn1 are shared among all three UBL species, K48-linked Ub_2_, and K11-linked Ub_2_. It is important to note that K48-linked Ub_3_ only exhibits ∼40% increased affinity for Rpn1^391-642^ compared with K48-linked Ub_2_ (24); therefore, we do not suspect that limited chain length is a factor.

It is unclear why the proteasomal degradation pathway exhibits so much redundancy. Numerous polyUb species can target substrates for degradation, with nearly endless combinations of possible linkage types, including branched and unbranched mixed-linkage chains. These polyUb chains may interact with UBL-UBA shuttle proteins that facilitate recognition by the proteasome, or they may bind directly to proteasomal receptors without the aid of shuttle proteins, albeit with weaker affinity in the case of Rpn1 (5, 6). Finally, once the substrate (tagged with polyUb and potentially a shuttle protein) reaches the proteasome, there are three receptors responsible for recognition: Rpn1, Rpn10, and Rpn13. Our results ostensibly reveal even greater redundancy in this system, as Rpn1 appears to contain multiple Ub/UBL-binding sites, none of which exhibit exclusivity toward one signal, thereby emphasizing the complex nature of polyUb and Ub-like signaling.

**Figure 6:**
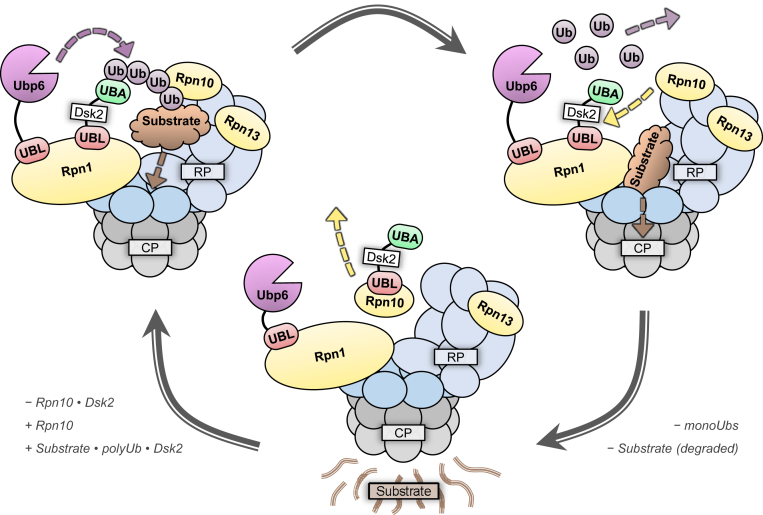
**Schematic of substrate processing by the proteasome.** (*Top left*) A substrate conjugated to polyUb⋅Dsk2 is anchored to the proteasome through two high-affinity interactions: Rpn1 binds the UBL domain of Dsk2, while Rpn10 binds polyUb. The substrate can then be positioned near the ring of ATPases (*dark blue*) and unfolded. Simultaneously, Ubp6 begins dismantling polyUb into Ub monomers. (*Top right*) The unfolded substrate is translocated into the CP where it is degraded into short peptides. As a result of diminished affinity, Rpn10 and Dsk2 disengage from monoUb, which can leave and be conjugated to another substrate. (*Bottom middle*) The substrate is completely destroyed and the CP is available to participate in another degradation event; however, Dsk2 must first be removed from Rpn1. Thus, extrinsic proteasomal subunit Rpn10 outcompetes Rpn1 for binding of Dsk2 and removes it, thereby allowing the degradation cycle to repeat. The RP is *blue*, the CP is *gray*, Ub is *purple*, UBL domains are *red*, UBA domains are *green*, Ubp6 is *magenta*, the substrate is *brown*, and proteasomal receptors (Rpn1, Rpn10, and Rpn13) are *yellow*.

These observations prompt important questions, such as: What is the purpose of “converting” a Ub signal into a UBL signal? Why do many UPS components exhibit redundant functionality? Unfortunately, these questions remain unanswered. Nevertheless, taking into account the results of our competition experiments involving both Rpn1 and Rpn10, we propose a model to describe how proteasomal recognition events may transpire (Fig. 6). We demonstrated that Rpn10 can compete with Rpn1 for binding to Dsk2-UBL, but not to Rad23-UBL; meanwhile, previous studies have shown that Rpn10 has weaker affinity for Dsk2-UBL than for K48-linked Ub_4_, but greater affinity for Dsk2-UBL than for monoUb (21). Taking the proteasome-destined complex of substrate⋅polyUb⋅Dsk2 as an example, Rpn1 preferentially recognizes the UBL domain of Dsk2, while Rpn10 preferentially recognizes long polyUb chains (Fig. 6, *top left*). Anchoring the polyUb⋅Dsk2 complex in multiple locations—a feature possible through recognition redundancy—allows the substrate to be tethered closely to the CP, thereby facilitating efficient entry into its central chamber. Concurrently, the catalytic domain of Rpn1-bound Ubp6 initiates cleavage of polyUb. Because the affinity of monoUb for Rpn1 and Rpn10 is relatively weak, the cleaved Ub monomers easily dissociate (Fig. 6, *top right*). However, Dsk2 needs to be removed from Rpn1 before another recognition event can occur. Conveniently, Rpn10 can outcompete Rpn1 for binding of Dsk2-UBL, thereby freeing Rpn1 (Fig. 6, bottom middle). This model fits with the existing description of Rpn10 as an extrinsic proteasomal receptor. Meanwhile, Rpn10 does not disturb Ubp6, allowing it to remain attached to Rpn1 and process another polyUb chain once the degradation cycle restarts.

Although not shown in this example, Rpn13 may perform the same role as Rpn10, as it also strongly associates with Dsk2-UBL and polyUb (9, 20). Perhaps the seemingly redundant presence of both Rpn10 and Rpn13 is beneficial such that when one receptor leaves or becomes unavailable, the other receptor can fill in, allowing the cycle to continue uninterrupted. It is important to emphasize that this model requires built-in redundancy, with multiple proteasomal receptors and multiple Ub/UBL-binding sites on Rpn1, to function at optimal efficiency.

This model, however, does not completely account for Rad23. Rad23 exhibits significantly stronger affinity for Rpn1 than that of polyUb and the other UBL-containing proteins (5, 6), while it cannot be removed by Rpn10. Based on published data (9, 20), Rad23 is also unlikely to be removed from Rpn1 by Rpn13. It is possible that the UPS allows for designation of high-priority signals, whereby substrates that require immediate degradation are tagged with Rad23. This would enable quick recognition of such substrates, allowing them to be processed before lower-priority substrates, like those tagged with polyUb or polyUb⋅Dsk2. It is worth noting that Rad23 has two UBA domains—unlike Dsk2 (one) and Ubp6 (zero)—which associate intramolecularly with the UBL domain, thereby engaging the same UBL surface that is involved in binding to Rpn1 and Rpn10 (20, 31, 32); thus, upon disassociation from polyUb, these UBAs may facilitate the release of Rad23 from the proteasome. Although further experiments are required to thoroughly characterize the binding mechanism of Rpn1 with its five major targets, our results suggest that the redundancy ingrained in the UPS may be a feature rather than a bug.

## Experimental procedures

### Protein preparation and purification

All proteins were expressed in *Escherichia coli* and purified as described previously: Ub monomers from *Homo sapiens* (33); Rad23, Dsk2, Ubp6, Ddi1, and their associated UBL domains from *Saccharomyces cerevisiae* (17, 18, 21, 34); Rpn10-UIM (residues 204–268, with a Q261Y mutation for quantification purposes) from *S. cerevisiae* (21, 34); and all Rpn1 (Rpn1^FL^, Rpn1^356-905^, Rpn1^391-642^, and Rpn1^391-642(AKAA)^) constructs from *S. cerevisiae* (5, 6, 17). The UBL domain constructs included the following residues (17, 21): 2–77 for Rad23-UBL (1–73 for competition studies with Rpn10-UIM); 2–77 for Dsk2-UBL; 2–81 for Ubp6-UBL; 1–80 for Ddi1-UBL. The UBL domain constructs also contained an N-terminal His-tag. The Dsk2-UBL construct contained a C-terminal extension (^78^LDLQPSLIS^87^) that does not affect its functionality (21). Uniprot accession numbers are as follows: P0CG48 (Ub); P32628 (Rad23); P48510 (Dsk2); P43593 (Ubp6); P40087 (Ddi1); P38886 (Rpn10); P38764 (Rpn1).

### Ubiquitin chain assembly

PolyUb chains were assembled *via* controlled-length enzymatic reactions (35, 36) along with domain-specific isotopic enrichment (33). Ub conjugating E2 enzymes Ube2S (37) (K11-specific) and Ube2K (aka E2-25K) (33) (K48-specific) were used to make respective linkages. Ub mutations were utilized to control polyUb length and architecture, with K11R/K48R/K63R mutations on the distal Ub and K63R/D77 mutations on the proximal Ub, since Ube2S makes a small fraction of K63-linkages (37).

### NMR spectroscopy

NMR experiments were performed on Bruker Avance III 600 MHz and 800 MHz spectrometers equipped with cryoprobes. Buffer conditions varied based on the stability of each Rpn1 construct: 20 mM sodium phosphate, 150 mM NaCl, 1 mM TCEP, 0.02% NaN_3_, 5–10% D_2_O, pH 6.8 for Rpn1^FL^; 50 mM Tris-Cl, 200 mM KCl, 5% glycerol, 5 mM β-mercaptoethanol, 0.02% NaN_3_, 5–10% D_2_O, pH 7.4 for Rpn1^356-905^; 50 mM HEPES, 50 mM KCl, 1 mM TCEP, 0.02% NaN_3_, 5–10% D_2_O, pH 7.6 for Rpn1^391-642^. The temperatures ranged from 21 °C to 25 °C.

Initial protein concentrations were between 30 μM and 250 μM, as detailed in the figure legends. Rpn1^FL^ precipitated at concentrations above 100 μM, while the other two constructs were more stable. Due to the instability of Rpn1^FL^, separate samples were prepared for each point in competition experiments. For Rpn1^356-905^ and Rpn1^391-642^, concentrated stocks were added stepwise to initial samples. A ^1^H-^15^N SOFAST-HMQC spectrum was recorded for each sample. NMR data were processed using TopSpin 3.0 (Bruker) and analyzed with Sparky (38).

Transverse (R_2_) ^15^N spin-relaxation rates for each residue were determined as described previously (39). Amide CSPs (Δδ) were calculated on a per-residue basis as follows:

(1) $$\Delta \delta =\sqrt{{\left(\Delta \delta_{H}\right)}^{2}+{\left(\Delta \delta_{N}/5\right)}^{2}}$$

where Δδ_H_ and Δδ_N_ correspond to differences in chemical shifts for the ^1^H and ^15^N resonances, respectively. The dissociation constant, K_d_, was quantified by fitting experimental CSPs at respective titration points to a single-site binding model using the in-house Matlab program Kdfit (40):

(2) $$\Delta \delta =\Delta \delta_{\max}\frac{\left[P_{t}\right]+\left[L_{t}\right]+K_{d}-\sqrt{{\left(\left[P_{t}\right]+\left[L_{t}\right]+K_{d}\right)}^{2}-4\left[P_{t}\right]\left[L_{t}\right]}}{2\left[P_{t}\right]}$$

where Δδ_max_ corresponds to the CSP value at saturation, [P_t_] is the total protein concentration, and [L_t_] is the total ligand concentration. K_d_ was treated as a global fitting parameter.

## Data availability

Data are available upon reasonable request from David Fushman: fushman@umd.edu.

## Supporting information

This article contains supporting information.

## Conflict of interest

The authors declare that they have no conflicts of interest with the contents of this article.

## Supporting information

Figures, Tables, References
