# Supplementary material for: Polyubiquitin and ubiquitin-like signals share common recognition sites on proteasomal subunit Rpn1
Source: J Biol Chem. 2021 Feb 20;296:100450. doi: 10.1016/j.jbc.2021.100450 (PMC8008175; doi:10.1016/j.jbc.2021.100450)
Supplement: Figures, Tables, References [file mmc1.pdf]

Supporting Information for:

Polyubiquitin and ubiquitin-like signals share common recognition sites on proteasomal subunit Rpn1

Andrew J. Boughton, Daoning Zhang, Rajesh K. Singh, and David Fushman*

Department of Chemistry and Biochemistry, Center for Biomolecular Structure and Organization, University of Maryland, College Park, MD 20742, USA

*Correspondence: David Fushman (fushman@umd.edu)

This PDF file includes:

Figures S1 to S10

Tables S1 to S2

Supporting Figures

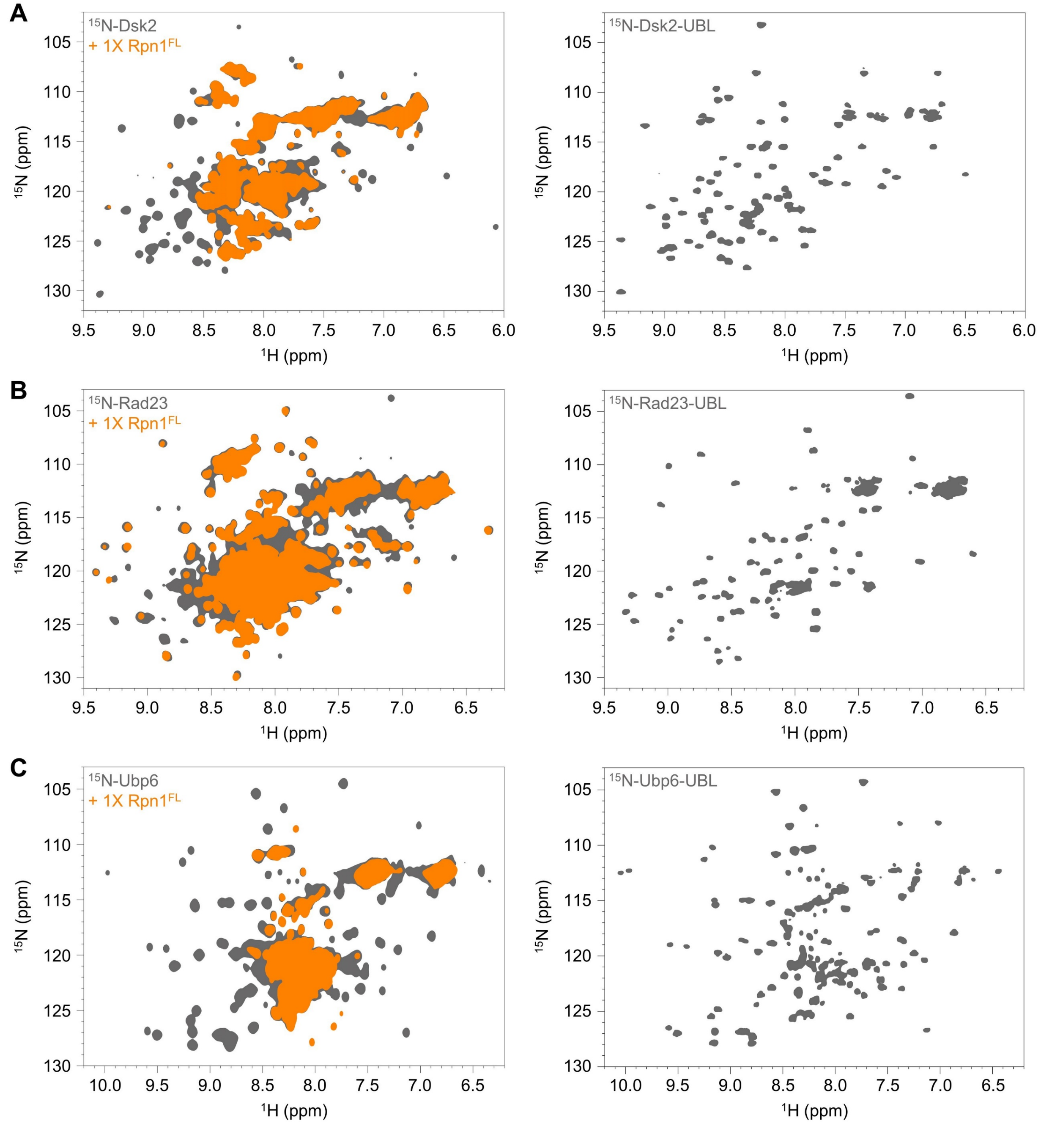

Figure S1: Rpn1 binds UBL-containing proteins. Overlaid ^{1}H-^{15}N NMR spectra of: (A, Left) 60 μM ^{15}N-Dsk2 (grey), 60 μM ^{15}N-Dsk2 plus 60 μM Rpn1^{FL} (orange); (B, Left) 70 μM ^{15}N-Rad23 (grey), 70 μM ^{15}N-Rad23 plus 70 μM Rpn1^{FL} (orange); (C, Left) 50 μM ^{15}N-Ubp6 (grey), 50 μM ^{15}N-Ubp6 plus 50 μM Rpn1^{FL} (orange). (A-C, Right) Respective spectra of each UBL domain by itself are included to facilitate visual comparison. Note that the UBL and UBA domains of Rad23 interact with each other (1-3); as a byproduct of this association, many of the signal positions in isolated Rad23-UBL differ from those of the UBL domain in full-length Rad23.

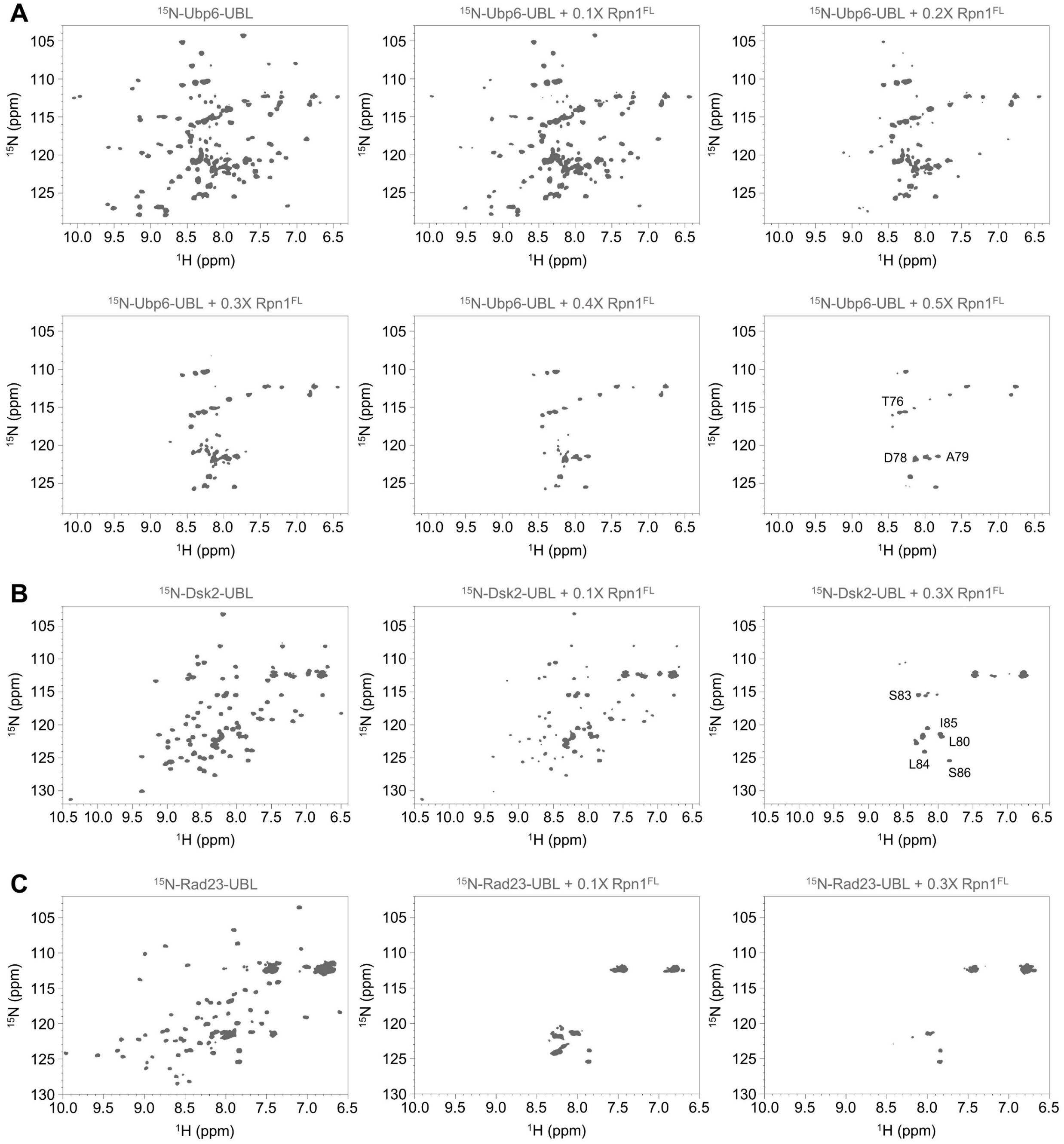

Figure S2: Widespread disappearance of ^{15}N-UBL NMR signals upon sub-stoichiometric addition of Rpn1. ^{1}H-^{15}N NMR spectra of: (A) 100 μM ^{15}N-Ubp6-UBL (top left), 100 μM ^{15}N-Ubp6-UBL plus 10 μM Rpn1^{FL} (top middle), 100 μM ^{15}N-Ubp6-UBL plus 20 μM Rpn1^{FL} (top right), 100 μM ^{15}N-Ubp6-UBL plus 30 μM Rpn1^{FL} (bottom left), 100 μM ^{15}N-Ubp6-UBL plus 40 μM Rpn1^{FL} (bottom middle), 100 μM ^{15}N-Ubp6-UBL plus 50 μM Rpn1^{FL} (bottom right); (B) 100 μM ^{15}N-Dsk2-UBL (left), 100 μM ^{15}N-Dsk2-UBL plus 10 μM Rpn1^{FL} (middle), 100 μM ^{15}N-Dsk2-UBL plus 30 μM Rpn1^{FL} (right); (C) 100 μM ^{15}N-Rad23-UBL (left), 100 μM ^{15}N-Rad23-UBL plus 10 μM Rpn1^{FL} (middle), 100 μM ^{15}N-Rad23-UBL plus 30 μM Rpn1^{FL} (right). Visible signals in the final spectra primarily correspond to flexible terminal residues and cloning artifacts.

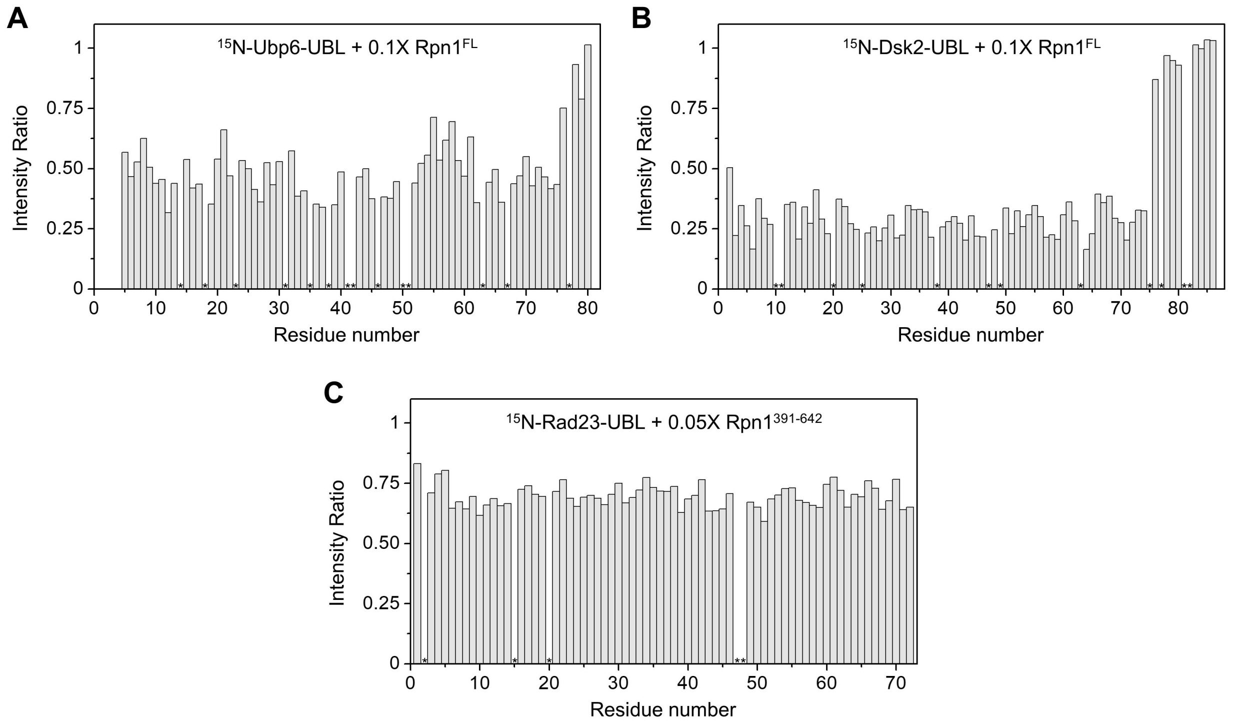

Figure S3: NMR signals of Ubp6-UBL, Dsk2-UBL, and Rad23-UBL attenuate non-specifically upon addition of Rpn1. Quantification of NMR signal intensity ratios on a per-residue basis for (A) 100 µM ^{15}N-Ubp6-UBL versus 100 µM ^{15}N-Ubp6-UBL plus 10 µM Rpn1^{FL} (spectra shown in Fig. S2A); (B) 100 µM ^{15}N-Dsk2-UBL versus 100 µM ^{15}N-Dsk2-UBL plus 10 µM Rpn1^{FL} (spectra shown in Fig. S2B); (C) 100 µM ^{15}N-Rad23-UBL versus 100 µM ^{15}N-Rad23-UBL plus 5 µM Rpn1$^{391\text{-}642}$. An intensity ratio of one indicates no signal disappearance, while an intensity ratio of zero indicates total signal disappearance. Residues denoted with an asterisk were not observed in the NMR spectra and were not included in analysis. Signals that did not exhibit substantial attenuation primarily correspond to flexible terminal residues and cloning artifacts. Intensity ratios were corrected for the dilution factor in each experiment. The data for Rad23-UBL is shown in the presence of Rpn1$^{391\text{-}642}$ because the corresponding NMR experiment with Rpn1^{FL} produced substantial signal attenuations (spectra shown in Fig. S2C), which prevented quantification of the signal intensity ratio.

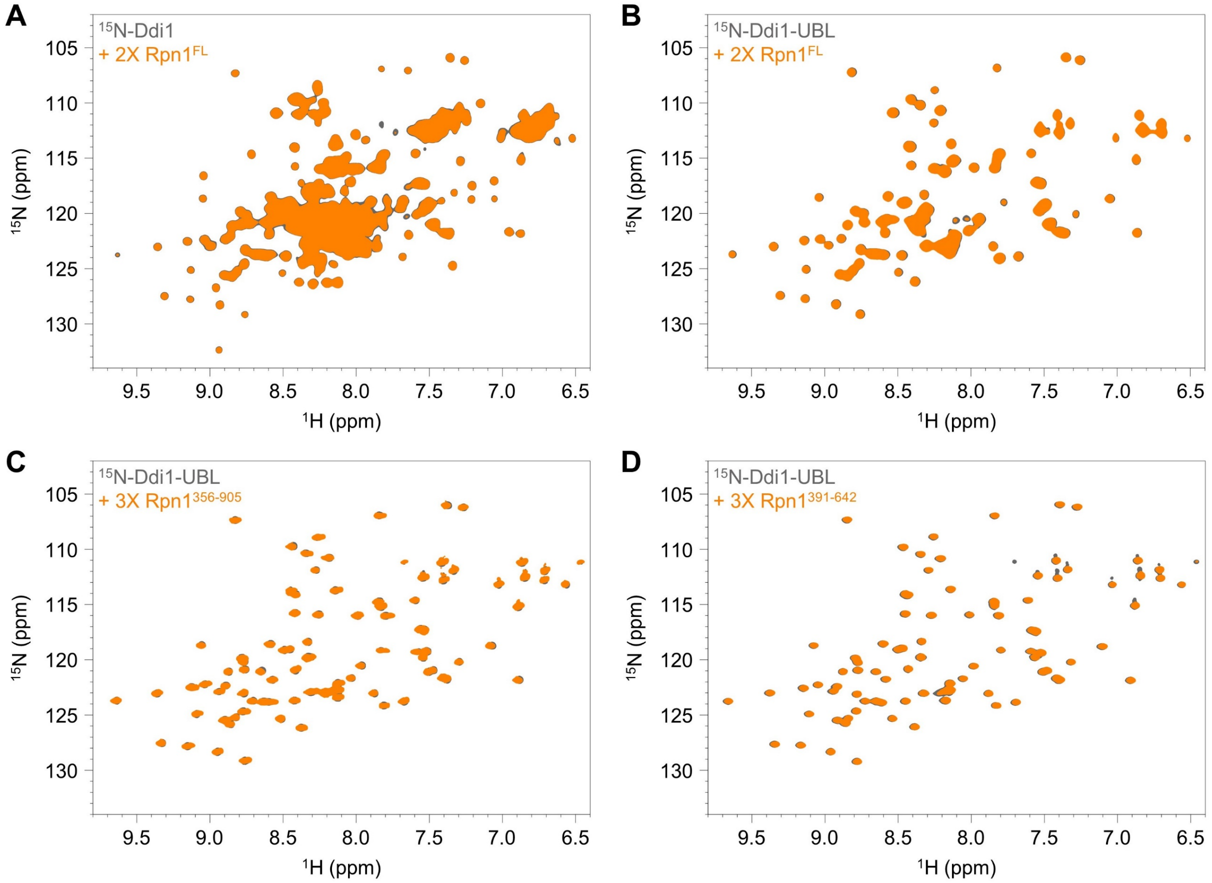

Figure S4: Rpn1 does not interact with Ddi1. Overlaid ^{1}H-^{15}N NMR spectra of: (A) 35 μM ^{15}N-Ddi1 (grey), 35 μM ^{15}N-Ddi1 plus 70 μM Rpn1^{FL} (orange); (B) 33 μM ^{15}N-Ddi1-UBL (grey), 33 μM ^{15}N-Ddi1-UBL plus 67 μM Rpn1^{FL} (orange); (C) 150 μM ^{15}N-Ddi1-UBL (grey), 150 μM ^{15}N-Ddi1-UBL plus 450 μM Rpn1$^{356\text{-}905}$ (orange); (D) 200 μM ^{15}N-Ddi1-UBL (grey), 200 μM ^{15}N-Ddi1-UBL plus 600 μM Rpn1$^{391\text{-}642}$ (orange).

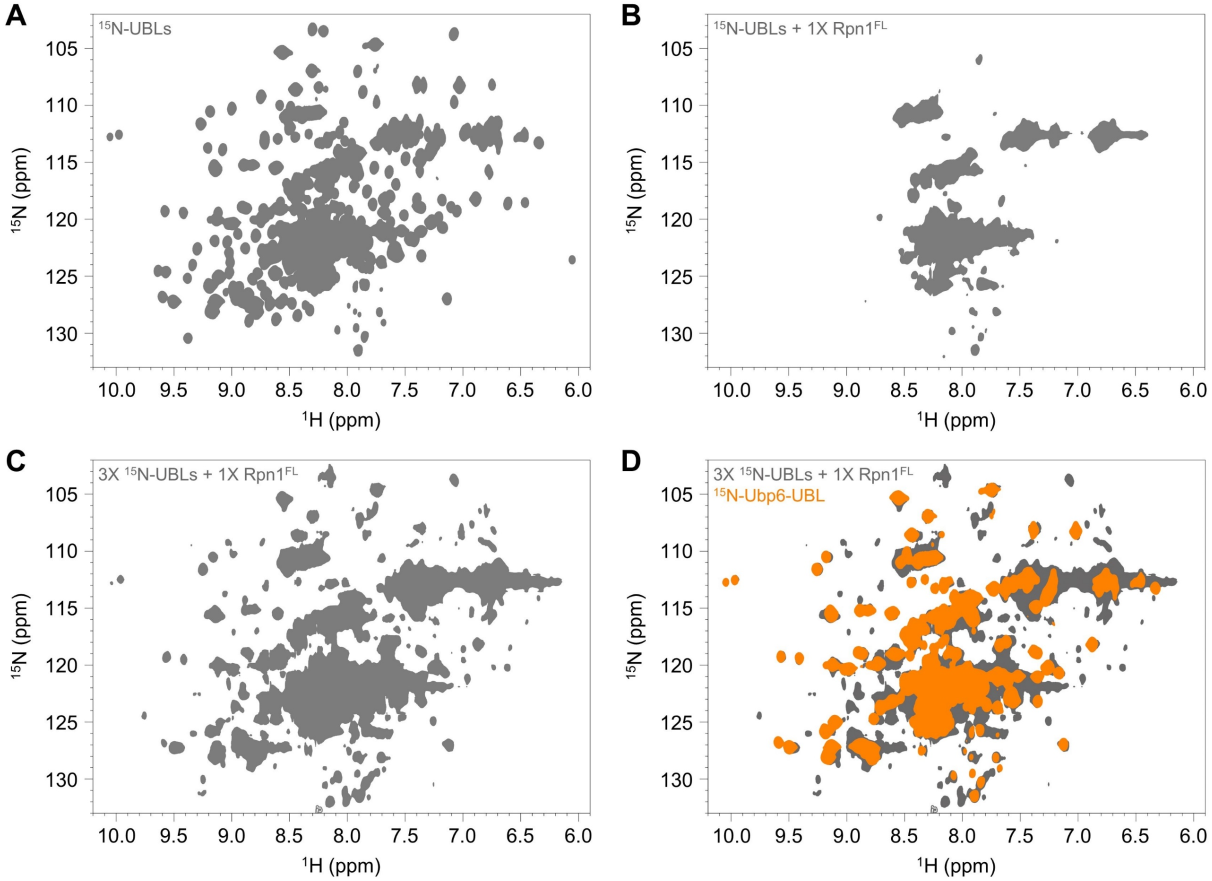

Figure S5: Rpn1 likely contains multiple UBL-binding sites. ^{1}H-^{15}N NMR spectra of: (A) an equimolar mixture of ^{15}N-Rad23-UBL, ^{15}N-Dsk2-UBL, and ^{15}N-Ubp6-UBL (50 μM of each ^{15}N-UBL); (B) 50 μM of each ^{15}N-UBL plus 50 μM $Rpn1^{FL}$; (C) 150 μM of each ^{15}N-UBL plus 50 μM $Rpn1^{FL}$; (D) 150 μM of each ^{15}N-UBL plus 50 μM $Rpn1^{FL}$ (grey) compared to ^{15}N-Ubp6-UBL by itself (orange).

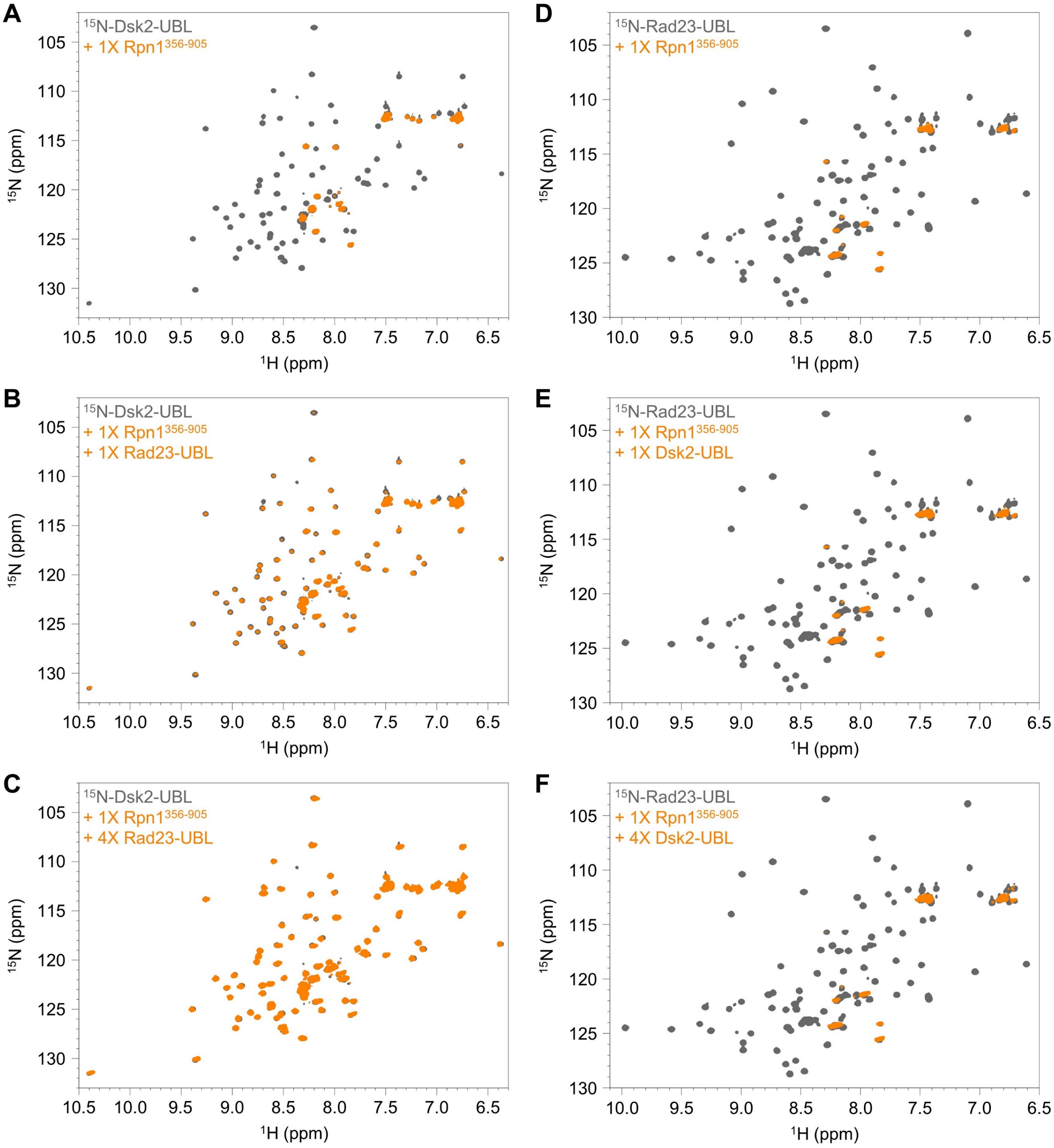

Figure S6: Rad23 outcompetes Dsk2 for binding to Rpn1 PC repeat region. Overlaid ^{1}H-^{15}N NMR spectra of: (A) ^{15}N-Dsk2-UBL (grey), ^{15}N-Dsk2-UBL plus 1X Rpn1$^{356-905}$ (orange); (B) ^{15}N-Dsk2-UBL (grey), ^{15}N-Dsk2-UBL plus 1X Rpn1$^{356-905}$ and 1X Rad23-UBL (orange); (C) ^{15}N-Dsk2-UBL (grey), ^{15}N-Dsk2-UBL plus 1X Rpn1$^{356-905}$ and 4X Rad23-UBL (orange); (D) ^{15}N-Rad23-UBL (grey), ^{15}N-Rad23-UBL plus 1X Rpn1$^{356-905}$ (orange); (E) ^{15}N-Rad23-UBL (grey), ^{15}N-Rad23-UBL plus 1X Rpn1$^{356-905}$ and 1X Dsk2-UBL (orange); (F) ^{15}N-Rad23-UBL (grey), ^{15}N-Rad23-UBL plus 1X Rpn1$^{356-905}$ and 4X Dsk2-UBL (orange). In (A-F), the concentration of the ^{15}N-enriched protein was 250 μM, such that a 1X molar equivalency corresponded to 250 μM and a 4X molar equivalency corresponded to 1 mM.

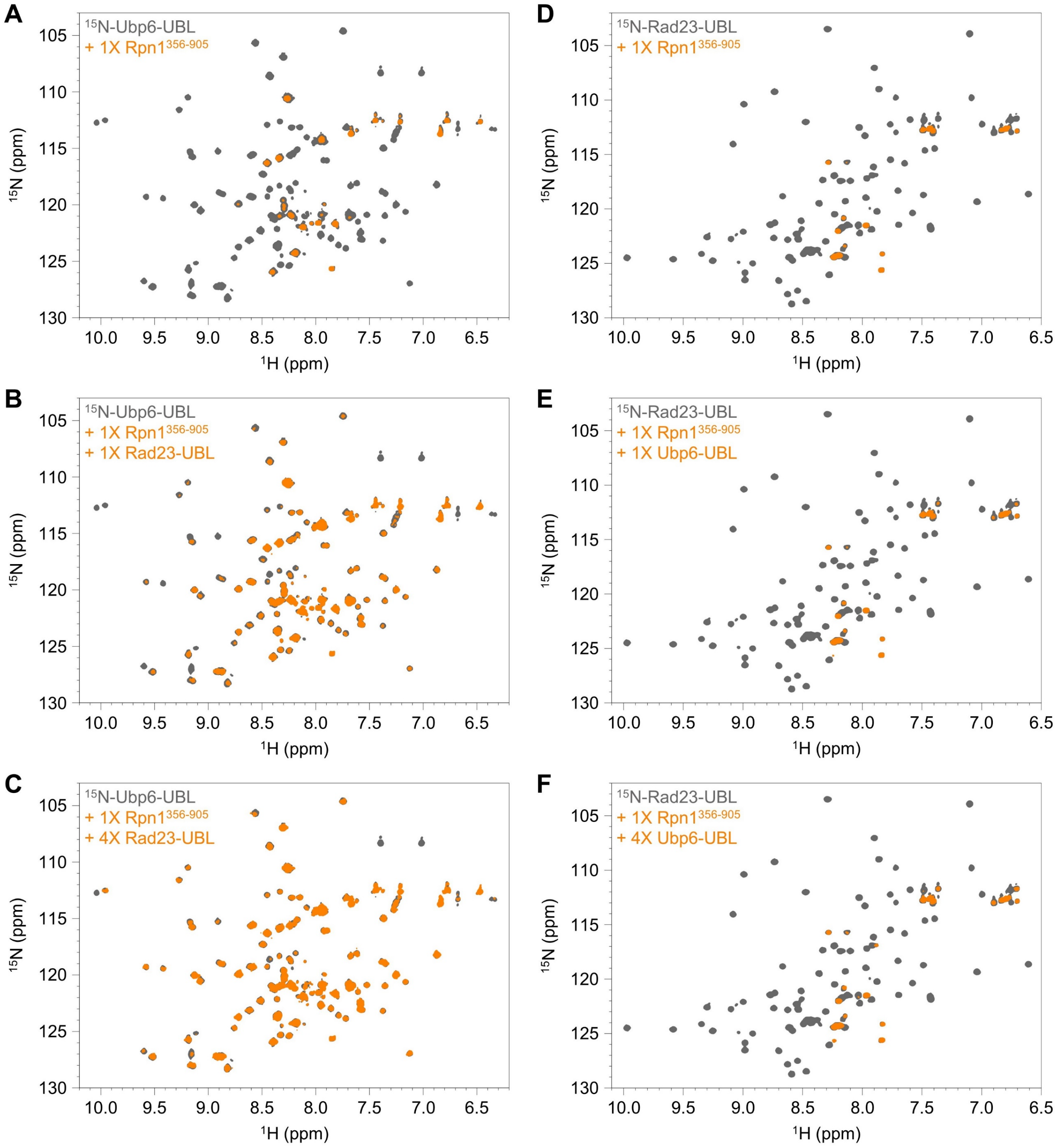

Figure S7: Rad23 outcompetes Ubp6 for binding to Rpn1 PC repeat region. Overlaid ^{1}H-^{15}N NMR spectra of: (A) ^{15}N-Ubp6-UBL (grey), ^{15}N-Ubp6-UBL plus 1X Rpn1$^{356\text{-}905}$ (orange); (B) ^{15}N-Ubp6-UBL (grey), ^{15}N-Ubp6-UBL plus 1X Rpn1$^{356\text{-}905}$ and 1X Rad23-UBL (orange); (C) ^{15}N-Ubp6-UBL (grey), ^{15}N-Ubp6-UBL plus 1X Rpn1$^{356\text{-}905}$ and 4X Rad23-UBL (orange); (D) ^{15}N-Rad23-UBL (grey), ^{15}N-Rad23-UBL plus 1X Rpn1$^{356\text{-}905}$ (orange); (E) ^{15}N-Rad23-UBL (grey), ^{15}N-Rad23-UBL plus 1X Rpn1$^{356\text{-}905}$ and 1X Ubp6-UBL (orange); (F) ^{15}N-Rad23-UBL (grey), ^{15}N-Rad23-UBL plus 1X Rpn1$^{356\text{-}905}$ and 4X Ubp6-UBL (orange). In (A-F), the concentration of the ^{15}N-enriched protein was 250 μM, such that a 1X molar equivalency corresponded to 250 μM and a 4X molar equivalency corresponded to 1 mM.

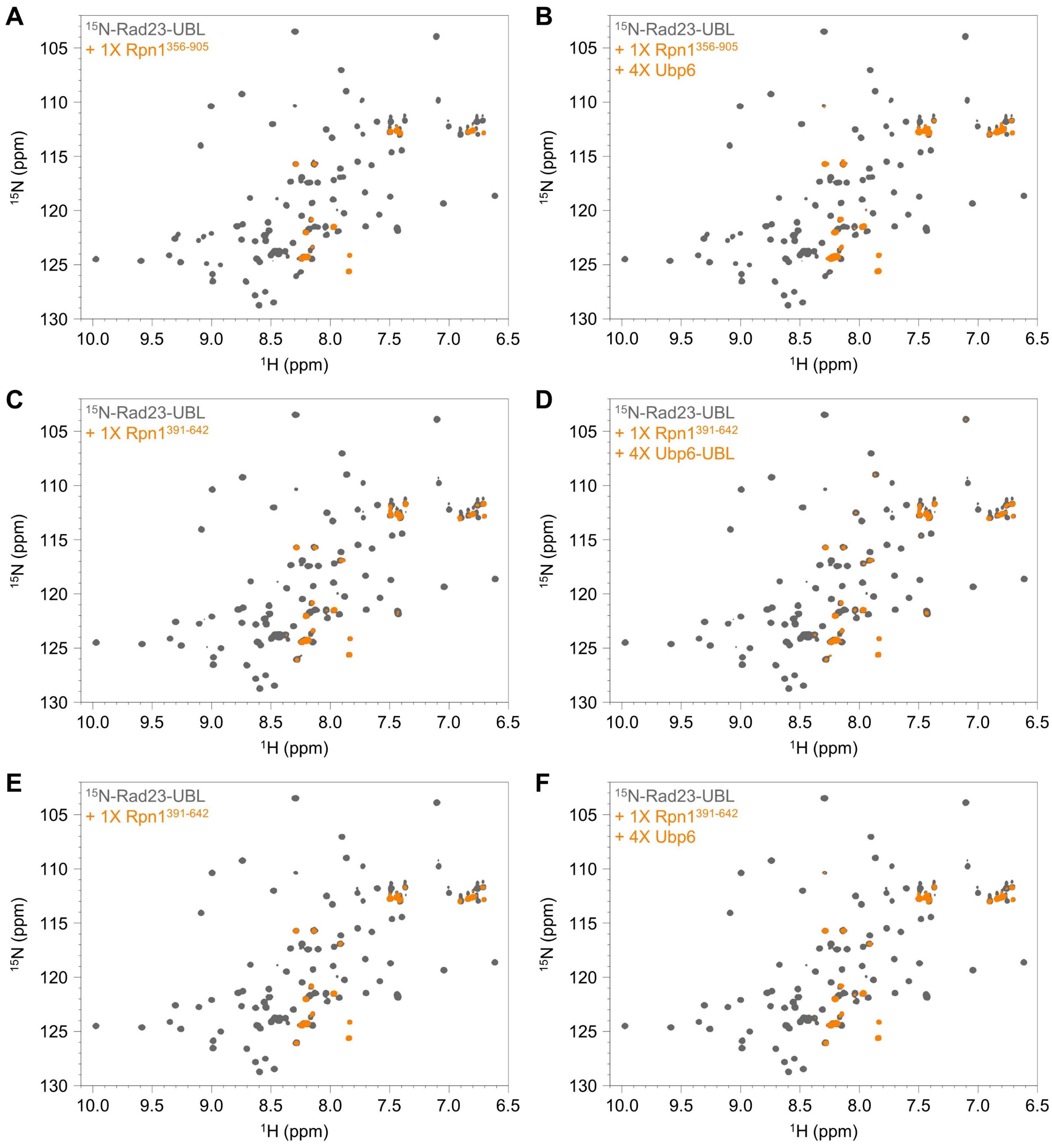

Figure S8: Neither Ubp6 nor Ubp6-UBL can outcompete Rad23 for binding to Rpn1 PC repeat region. Overlaid ^{1}H-^{15}N NMR spectra of: (A) ^{15}N-Rad23-UBL (grey), ^{15}N-Rad23-UBL plus 1X Rpn1$^{356\text{-}905}$ (orange); (B) ^{15}N-Rad23-UBL (grey), ^{15}N-Rad23-UBL plus 1X Rpn1$^{356\text{-}905}$ and 4X Ubp6 (orange); (C) ^{15}N-Rad23-UBL (grey), ^{15}N-Rad23-UBL plus 1X Rpn1$^{391\text{-}642}$ (orange); (D) ^{15}N-Rad23-UBL (grey), ^{15}N-Rad23-UBL plus 1X Rpn1$^{391\text{-}642}$ and 4X Ubp6-UBL (orange); (E) ^{15}N-Rad23-UBL (grey), ^{15}N-Rad23-UBL plus 1X Rpn1$^{391\text{-}642}$ (orange); (F) ^{15}N-Rad23-UBL (grey), ^{15}N-Rad23-UBL plus 1X Rpn1$^{391\text{-}642}$ and 4X Ubp6 (orange). In (A-F), the concentration of the ^{15}N-enriched protein was 200 μM, such that a 1X molar equivalency corresponded to 200 μM and a 4X molar equivalency corresponded to 800 μM.

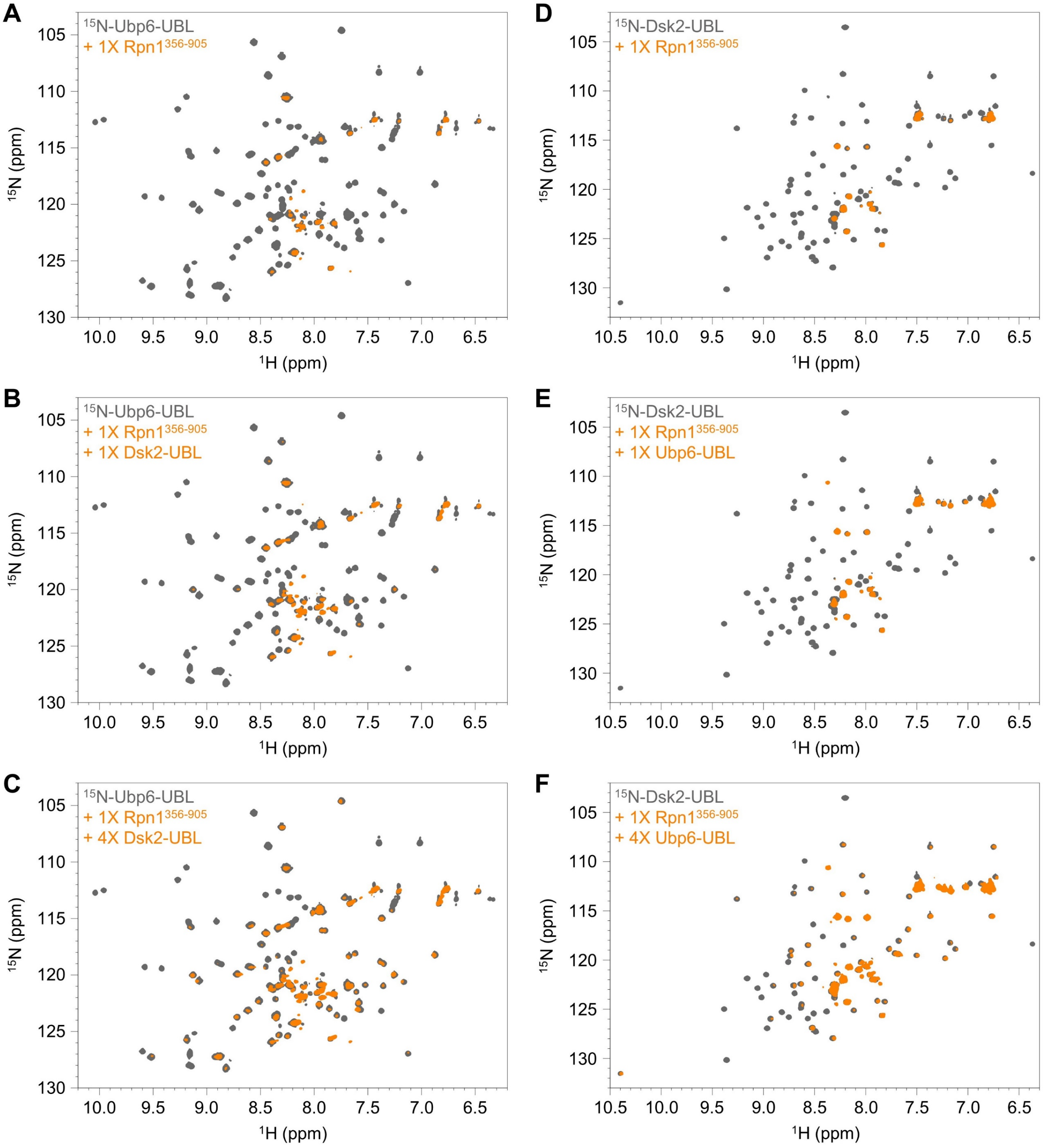

Figure S9: Ubp6 and Dsk2 compete with each other for binding to Rpn1 PC repeat region. Overlaid ^{1}H-^{15}N NMR spectra of: (A) ^{15}N-Ubp6-UBL (grey), ^{15}N-Ubp6-UBL plus 1X Rpn1$^{356\text{-}905}$ (orange); (B) ^{15}N-Ubp6-UBL (grey), ^{15}N-Ubp6-UBL plus 1X Rpn1$^{356\text{-}905}$ and 1X Dsk2-UBL (orange); (C) ^{15}N-Ubp6-UBL (grey), ^{15}N-Ubp6-UBL plus 1X Rpn1$^{356\text{-}905}$ and 4X Dsk2-UBL (orange); (D) ^{15}N-Dsk2-UBL (grey), ^{15}N-Dsk2-UBL plus 1X Rpn1$^{356\text{-}905}$ (orange); (E) ^{15}N-Dsk2-UBL (grey), ^{15}N-Dsk2-UBL plus 1X Rpn1$^{356\text{-}905}$ and 1X Ubp6-UBL (orange); (F) ^{15}N-Dsk2-UBL (grey), ^{15}N-Dsk2-UBL plus 1X Rpn1$^{356\text{-}905}$ and 4X Ubp6-UBL (orange). In (A-F), the concentration of the ^{15}N-enriched protein was 250 µM, such that a 1X molar equivalency corresponded to 250 µM and a 4X molar equivalency corresponded to 1 mM.

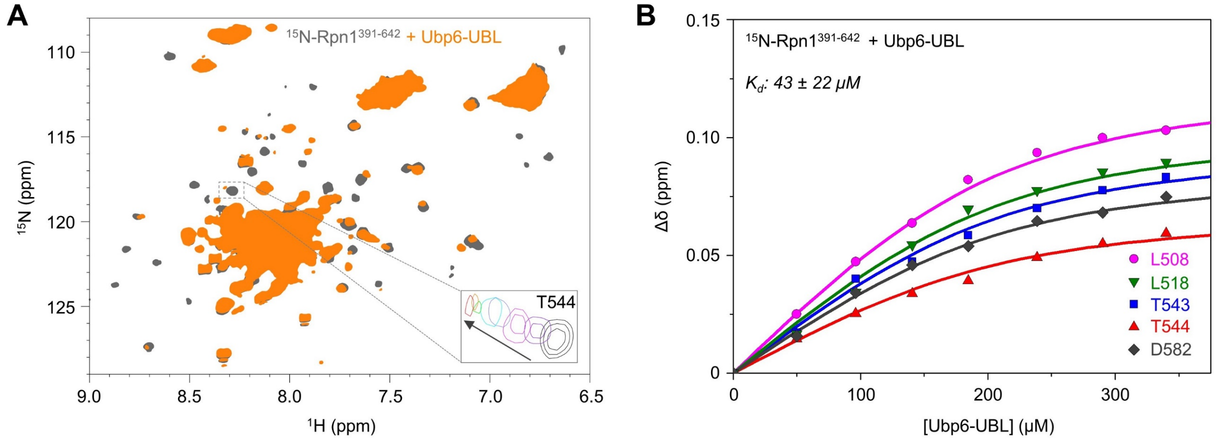

Figure S10: Rpn1$^{391\text{-}642}$ binds Ubp6-UBL with expected affinity. (A) Overlaid ^{1}H-^{15}N NMR spectra of ^{15}N-Rpn1$^{391\text{-}642}$ (grey) and ^{15}N-Rpn1$^{391\text{-}642}$ plus equimolar Ubp6-UBL (orange); as a representative example, the signal of T544 after each addition of Ubp6-UBL is shown in the inset. (B) NMR titration CSPs (Δδ) for select residues in ^{15}N-Rpn1$^{391\text{-}642}$ as a function of Ubp6-UBL concentration. The curves are a result of fitting the experimental data to a single-site binding model.

Supporting Tables

Table S1: Compilation of affinities of UBL domains for Rpn1 fragments.

	**Rpn1$^{391\text{-}642}$ K_d (μM)**	**Rpn1$^{412\text{-}625}$ K_d (μM)**	**Rpn1$^{391\text{-}642(AKAA)}$ K_d (μM)**
**Rad23-UBL**	-	0.064 ± 0.025 [b]	-
**Dsk2-UBL**	22 ± 12 [a]	-	-
**Ubp6-UBL**	40 ± 31 [a]<br>43 ± 22 [c]	-	36 ± 21 [c]

[a] Reported previously (4).
[b] Reported previously (5).
[c] Reported here.

Table S2: Published and predicted characteristics of the kinetics of $Rpn1^{FL}$ binding to full-length UBL-containing proteins and to the respective UBL domains.

	K_d (μM)	k_{on} ($M^{-1}s^{-1}$)	k_{off} (s^{-1})	$1/k_{off}$ (s) [c]
**Rad23** [a]	3.65 ± 0.09	$2.02 \pm 0.56 \times 10^4$	0.074 ± 0.022	13.5
**Dsk2** [a]	12.10 ± 0.90	$1.37 \pm 0.04 \times 10^4$	0.165 ± 0.007	6.1
**Ubp6** [a]	1.92 ± 0.26	$3.53 \pm 0.73 \times 10^4$	0.067 ± 0.006	14.9
**Rad23-UBL** [a]	3.59 ± 0.69	5.37×10^4 [b]	0.19 [b]	5.3
**Dsk2-UBL** [a]	11.69 ± 0.87	5.37×10^4 [b]	0.63 [b]	1.6
**Ubp6-UBL** [a]	2.23 ± 0.24	$5.37 \pm 0.54 \times 10^4$	0.120 ± 0.013	8.3

[a] Experimental SPR data reported previously (6). Note that full-length Ubp6 contains two Rpn1-binding regions: the UBL domain and the UBP domain. Only the SPR measurements pertaining to the UBL domain of Ubp6 are included here.

[b] Because experimental association (k_{on}) and dissociation (k_{off}) rates for isolated UBL domains are only available for Ubp6-UBL, the dissociation rates for Rad23-UBL and Dsk2-UBL were estimated based on their K_d values ($k_{off} = K_d \times k_{on}$) and the assumption that all three UBL domains exhibit similar sizes, structures, and on-rates; thus, $k_{on} = 5.37 \times 10^4$ M^{-1} s^{-1} was assumed for Rad23-UBL and Dsk2-UBL. Alternatively, the k_{off} values measured for full-length Rad23 and Dsk2 should provide an estimate for k_{off} of the respective UBL domains, because the UBL domain is the sole moiety in Rad23 and Dsk2 responsible for binding Rpn1.

[c] For full-length proteins (Rad23, Dsk2, Ubp6), the observed residence time (estimated as $1/k_{off}$) in complex with $Rpn1^{FL}$ greatly exceeds the characteristic time of the NMR measurement and data collection period (100-150 ms for the pulse sequence and acquisition). Regardless of whether the off-rate of each UBL domain is estimated or measured for the full-length protein, these k_{off} values correspond to a residence time in the Rpn1-bound state greater than one second. Thus, both the full-length proteins and the UBL domains that are in the Rpn1-bound state do not have enough time to dissociate from Rpn1 during the characteristic NMR experiment time. Consequently, each NMR experiment should exhibit two species, one in the free state and one in the Rpn1-bound state; due to the slow off-rates, these species do not have sufficient time to efficiently interconvert.

Supporting References

1. Rao, H., and Sastry, A. (2002) Recognition of specific ubiquitin conjugates is important for the proteolytic functions of the ubiquitin-associated domain proteins Dsk2 and Rad23. *J Biol Chem* **277**, 11691-11695
2. Walters, K. J., Lech, P. J., Goh, A. M., Wang, Q., and Howley, P. M. (2003) DNA-repair protein hHR23a alters its protein structure upon binding proteasomal subunit S5a. *Proc Natl Acad Sci U S A* **100**, 12694-12699
3. Goh, A. M., Walters, K. J., Elsasser, S., Verma, R., Deshaies, R. J., Finley, D., and Howley, P. M. (2008) Components of the ubiquitin-proteasome pathway compete for surfaces on Rad23 family proteins. *BMC Biochem* **9**, 4
4. Chojnacki, M., Mansour, W., Hameed, D. S., Singh, R. K., El Oualid, F., Rosenzweig, R., Nakasone, M. A., Yu, Z., Glaser, F., Kay, L. E., Fushman, D., Ovaa, H., and Glickman, M. H. (2017) Polyubiquitin-Photoactivatable Crosslinking Reagents for Mapping Ubiquitin Interactome Identify Rpn1 as a Proteasome Ubiquitin-Associating Subunit. *Cell Chem Biol* **24**, 443-457.e446
5. Shi, Y., Chen, X., Elsasser, S., Stocks, B. B., Tian, G., Lee, B. H., Zhang, N., de Poot, S. A., Tuebing, F., Sun, S., Vannoy, J., Tarasov, S. G., Engen, J. R., Finley, D., and Walters, K. J. (2016) Rpn1 provides adjacent receptor sites for substrate binding and deubiquitination by the proteasome. *Science* **351**, aad94211-aad942110
6. Rosenzweig, R., Bronner, V., Zhang, D., Fushman, D., and Glickman, M. H. (2012) Rpn1 and Rpn2 coordinate ubiquitin processing factors at proteasome. *J Biol Chem* **287**, 14659-14671
